# Long-Range Resonant Charge Transport through Open-Shell Donor–Acceptor Macromolecules

**DOI:** 10.1021/jacs.4c18150

**Published:** 2025-05-01

**Authors:** Shaocheng Shen, Mehrdad Shiri, Paramasivam Mahalingam, Chaolong Tang, Tyler Bills, Alexander J. Bushnell, Tanya A. Balandin, Leopoldo Mejía, Haixin Zhang, Bingqian Xu, Ignacio Franco, Jason D. Azoulay, Kun Wang

**Affiliations:** † Department of Chemistry, 5452 University of Miami, Coral Gables, Florida 33146, United States; ‡ Department of Physics, University of Miami, Coral Gables, Florida 33146, United States; § School of Chemistry and Biochemistry, School of Materials Science and Engineering, Center for Organic Photonics and Electronics, 1372 Georgia Institute of Technology, Atlanta, Georgia 30332, United States; ∥ Department of Physics and astronomy, 5547 Mississippi State University, Mississippi State, Mississippi 39762, United States; ⊥ Departamento de Física y Astronomía, Facultad de Ciencias Exactas, 28087 Universidad Andrés Bello, Santiago 837-0136, Chile; # Single Molecule Study Laboratory, College of Engineering and Nanoscale Science and Engineering Center, 1355 University of Georgia, Athens, Georgia 30602, United States; ∇ Department of Chemistry, 6927 University of Rochester, Rochester, New York 14627, United States; ○ Department of Physics, University of Rochester, Rochester, New York 14627, United States

## Abstract

A grand challenge in molecular electronics is the development of molecular materials that can facilitate efficient long-range charge transport. Research spanning more than two decades has been fueled by the prospects of creating a new generation of miniaturized electronic technologies based on molecules whose synthetic tunability offers tailored electronic properties and functions unattainable with conventional electronic materials. However, current design paradigms produce molecules that exhibit off-resonant transport under low bias, which limits the conductance of molecular materials to unsatisfactorily low levels—several orders of magnitude below the conductance quantum 1 *G*_0_—and often results in an exponential decay in conductance with length. Here, we demonstrate a chemically robust, air-stable, and highly tunable molecular wire platform comprised of open-shell donor–acceptor macromolecules that exhibit remarkably high conductance close to 1 *G*_0_ over a length surpassing 20 nm under low bias, with no discernible decay with length. Single-molecule transport measurements and *ab initio* calculations show that the ultralong-range resonant transport arises from extended π-conjugation, a narrow bandgap, and diradical character, which synergistically enables excellent alignment of frontier molecular orbitals with the electrode Fermi energy. The implementation of this long-sought-after transport regime within molecular materials offers new opportunities for the integration of manifold properties within emerging nanoelectronic technologies.

## Graphical abstract

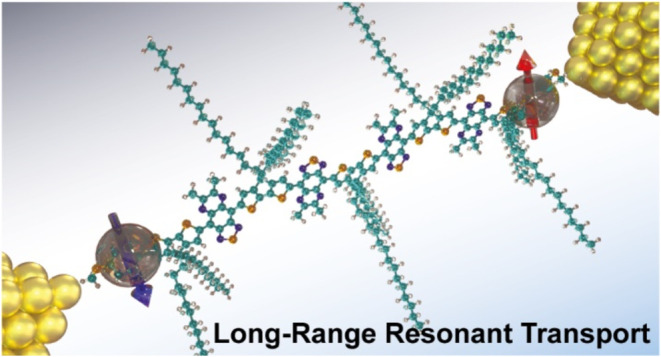

## Introduction

Creating highly conducting molecular materials capable of efficient long-range charge transport is central to the development of emerging molecular-scale technologies, including electronics,^1–3^ energy conversion,^4–6^ sensing,^7,8^ spintronics^9,10^ and quantum information science.^11,12^ To establish a molecular-level understanding of charge transport through discrete molecules, the interdisciplinary scientific community has developed experimental techniques capable of fabricating single-molecule junctions (SMJs) that enable direct characterization of charge transport through a molecular backbone.^13,14^ Numerous SMJ studies have elucidated how molecular design governs electron transmission through molecular systems, demonstrating that organic π-conjugated molecules yield higher electrical conductance than their nonconjugated counterparts. In π-conjugated materials, charge transport at the single-molecule level is predominantly governed by coherent and off-resonant electron tunneling in the low-bias regime.^15,16^ However, the intrinsic nature of off-resonant transport limits the conductance of a molecular backbone to values that are several orders of magnitude lower than the conductance quantum 1 *G*_0_ (where *G*_0_ = 2 e^2^/*h* represents the conductance for a metallic quantum point contact). Furthermore, in the off-resonant tunneling regime, an increase in molecular length results in an exponential conductance decay following *G* = *G*_*C*_ e^–β*L*^,^17^ where β and *L* are the conductance attenuation factor and molecular length, respectively, leading to insulating behavior for longer molecules. These unfavorable transport characteristics constitute a formidable challenge that hinders the utilization, scalability, and compatibility of molecular materials in nanoelectronic devices.

To meet the demands of emerging technologies and rival the performance of conventional electronic wires in modern integrated circuits, an ideal molecular wire must be air-stable, chemically robust, operate at low bias, possess quasi-metallic behavior (*i.e.*, conductance close to 1 *G*_0_), and exhibit no conductance attenuation with length, properties that have yet to be realized. Diverse chemical approaches to achieve high conductance in single molecules have explored various design strategies, including extended π-conjugation,^18–20^ noncovalent interactions,^21,22^ and more recently, endowing diradicaloid character in analogy to strategies for quasi-metallic polymers following the Su–Schrieffer–Heeger (SSH) model.^23–26^ Despite improvements in conductance in oxidized, reduced, and open-shell forms of π-conjugated molecules, several major challenges have not been overcome, including low conductance values from ∼10^–1^ to 10^–4^ *G*_0_ and high chemical instability of these molecular frameworks, which inhibit their practical utilization in ambient conditions. This is compounded by the absence of design guidelines to access materials with intrinsic and interdependent properties, including extensive π-conjugation, high stability, low static and dynamic disorder, and small reorganization energies, attributes that are theorized to promote long-range resonant charge transport.^27,28^ Consequently, molecular wires capable of facilitating quasi-metallic charge transport across length scales of tens of nanometers have been unattainable despite their fundamental and practical significance.

Here, we demonstrate chemically robust and air-stable molecular wires comprised of charge-neutral, open-shell donor–acceptor (DA) macromolecules that exhibit remarkably high single-molecule conductance close to 1 *G*_0_ across a molecular backbone length exceeding 20 nm, a previously uncharted length scale in molecular electronics. These macromolecules are of facile synthesis, and their ultrahigh conductance values and ultralong transport lengths exceed those of the best-performing organic materials. As demonstrated using a homologous series of molecules with increasing length, scanning tunneling microscopy break junction (STM-BJ) measurements, and density functional theory (DFT) calculations, the transport phenomena in this macromolecular platform are attributed to long-range resonant transport that arises from the synergistic combination of extended π-conjugation, a narrow bandgap, and open-shell electronic structure (*i.e.*, diradicaloid character) that introduces highly conducting states at midgap. These features synergistically facilitate excellent alignment of frontier molecular orbitals (FMOs) with the electrode Fermi energy (*E*_F_), enabling resonant coherent electron transport in the low bias regime. Additionally, we demonstrate that through mechanical modulation, the conductance of a 20 nm long macromolecular wire can be enhanced to 1 *G*_0_, representing the first observation of ballistic transport in molecules at the tens of nanometers length scale.

## Results and Discussion

Figure 1 displays the structure of the open-shell macromolecular framework used in this study, poly­[4-(4,4-dihexadecyl-4*H*-cyclopenta­[2,1-*b*:3,4-*b*′]­dithiophen-2-yl)-*alt*-6,7-dimethyl-[1,2,5]­thiadiazolo­[3,4-*g*]­quinoxaline]. Salient design features include the 4*H*-cyclopenta­[2,1-*b*:3,4-*b*′]­dithiophene (CPDT) donor which stabilizes the highest occupied molecular orbital (HOMO) and strong thiadiazoloquinoxaline (TQ) acceptor that lowers the lowest unoccupied molecular orbital (LUMO) and promotes strong electronic correlations that enable a narrowing of the bandgap and quinoidal bonding patterns. These features form and stabilize unpaired spins within extended π-conjugated structures and donor–acceptor conjugated polymers.^29–31^ This DA framework results in extended π-conjugation, a rigid backbone, and open-shell electronic structures in which valence α- and β-spins occupy singly occupied molecular orbitals (SOMOs) that are extensively delocalized within the conjugated backbone, molecular features known to promote improved long-range transport and high chemical stability.^26,28^

**Figure 1:**
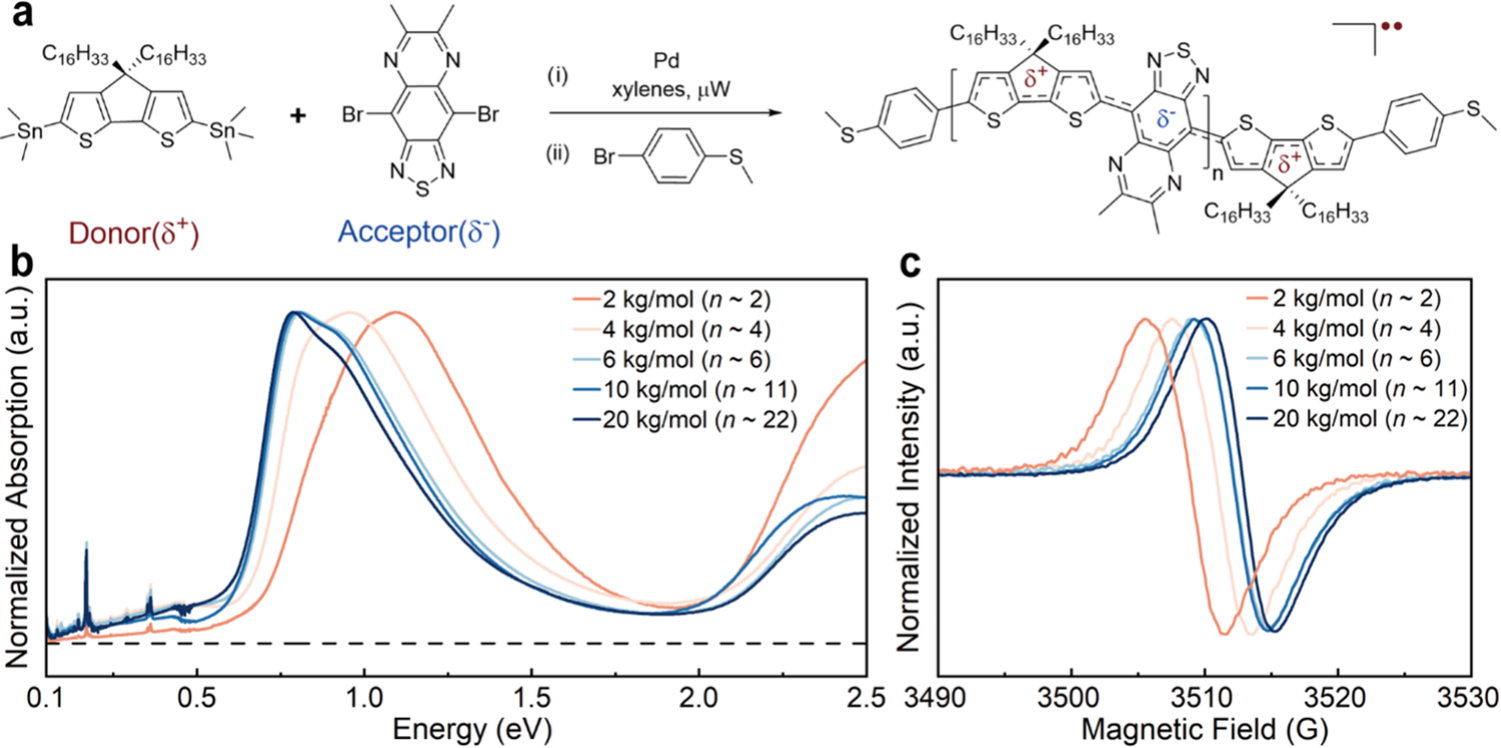
Synthesis of the open-shell donor–acceptor macromolecules and solid-state properties. (a) The molecular building blocks and Stille cross-coupling polymerization used to synthesize the end-functionalized macromolecules. (b) Absorption spectra of thin films spin-coated from chlorobenzene onto quartz and KBr substrates. (c) EPR (X-band) spectra of solids at room temperature.

To investigate the impact of chain length (or repeat unit *n*) on the electronic structure and conductance of the macromolecules, we designed a synthetic approach to access end-functionalized variants compatible with STM-BJ measurements (Figure 1a). We utilized a microwave-mediated Stille cross-coupling methodology between (4,4-dihexadecyl-4*H*-cyclopenta­[2,1-*b*:3,4-*b*′]­dithiophene-2,6-diyl)­bis­(trimethylstannane) and 4,9-dibromo-6,7-dimethyl-[1,2,5]­thiadiazolo­[3,4-*g*]­quinoxaline (Figure 1a). Using Pd­(PPh_3_)_4_ as the catalyst and modifying the stoichiometry using a Carothers approach to control the number-average molecular weight (*M_n_*) enabled rapid access to macromolecules with *M_n_* that span a wide range from *M_n_* ∼ 2 kg/mol (*n* ∼ 2)–20 kg/mol (*n* ∼ 22). In this approach, the donor monomer in stoichiometric excess gives the degree of polymerization (*X_n_*) for a given fractional monomer conversion (*p*), (*i.e.*, *X_n_* = (1 + *r*)/(1 + *r* – 2*rp*)), where *r* is the stoichiometric ratio of monomers. This results in a macromolecular chain end-functionalized with the CPDT donor and reactive–SnMe_3_ functionality. The polymerization reaction was followed by *in situ* end-group functionalization with 4-bromothioanisole resulting in chain ends functionalized with methylthiobenzene units known to bind to gold electrodes (Figure 1a). Additional details can be found in the Supporting Information and Figures S1–S8. As shown in Figure 1b, these macromolecules exhibit progressively narrow bandgaps, with absorption maxima (λ_max_) spanning 1.13–1.58 μm, and measurable absorbance extending beyond the midwavelength infrared for higher molecular weight materials (Figures S9–S14 and Table S1). These features are attributable to more extensive π-conjugation and a higher degree of electronic coherence as a function of chain length. The concomitant increase in the low-energy absorption correlates with an increase in diradical character and associated near-degenerate partially occupied orbital manifold.^29–31^ Room temperature continuous-wave electron paramagnetic resonance (EPR) spectra of the samples show broad single peaks with *g-*factors centered at 2.0038–2.0039, consistent with organic delocalized diradicals having little spin–orbit coupling from heteroatoms along the π-conjugated backbone (Figure 1c). A decrease in the line widths from 6.3 to 5.0 gauss across the series is consistent with less inhomogeneous broadening at higher molecular weights in accordance with each spin delocalized over more nuclei and a decrease in the net hyperfine coupling. Variable temperature EPR measurements of the longest 20 kg/mol macromolecule give a nearly degenerate high-to-low spin energy gap (Δ*E*_ST_) of 6.48 × 10^–3^ kcal/mol, consistent with previous reports (Figure S15).^29^

We characterized the single-molecule conductance of the macromolecules under an applied bias of 200 mV using the STM-BJ technique (Figure 2a), as detailed in the Supporting Information. The robust stability of the macromolecules enabled conductance measurements in air under ambient conditions. We first investigated a homologous series of open-shell macromolecules with varying *M*_*n*_ to interrogate their conductance and the impact of molecular length. Figure 2b illustrates the one-dimensional (1D) conductance histograms of macromolecules with target *M*_*n*_ of 2, 4, 6, 10, and 20 kg/mol (average *n* ∼ 2, 4, 6, 11, and 22, respectively). The most probable conductance for each macromolecule was determined by fitting the 1D histogram peak with a Gaussian fitting. The 1D histograms of all macromolecules display a single dominant conductance peak that is remarkably high. Specifically, the conductance values of the macromolecules with *M*_*n*_ of 2, 4, 6, 10, and 20 kg/mol are 10^–1.25^ *G*_0_, 10^–1.66^ *G*_0_, 10^–1.41^ *G*_0_, 10^–0.46^ *G*_0_, and 10^–0.79^ *G*_0_, respectively (Table S2). We statistically analyzed the molecular plateau length of the measured conductance *vs* displacement traces, as shown in Figure 2c. Representative traces and two-dimensional (2D) conductance *vs* displacement histograms are illustrated in Figure 2d. These histograms reveal that the conductance plateau length consistently increases in proportion to the molecular length (*i.e.*, calculated sulfur-to-sulfur lengths of these macromolecules) (Table S2). The measured plateau lengths are 1.7, 5.4, 7.5, 10.6, and 19.4 nm for 2, 4, 6, 10, and 20 kg/mol macromolecular wires, respectively.

**Figure 2:**
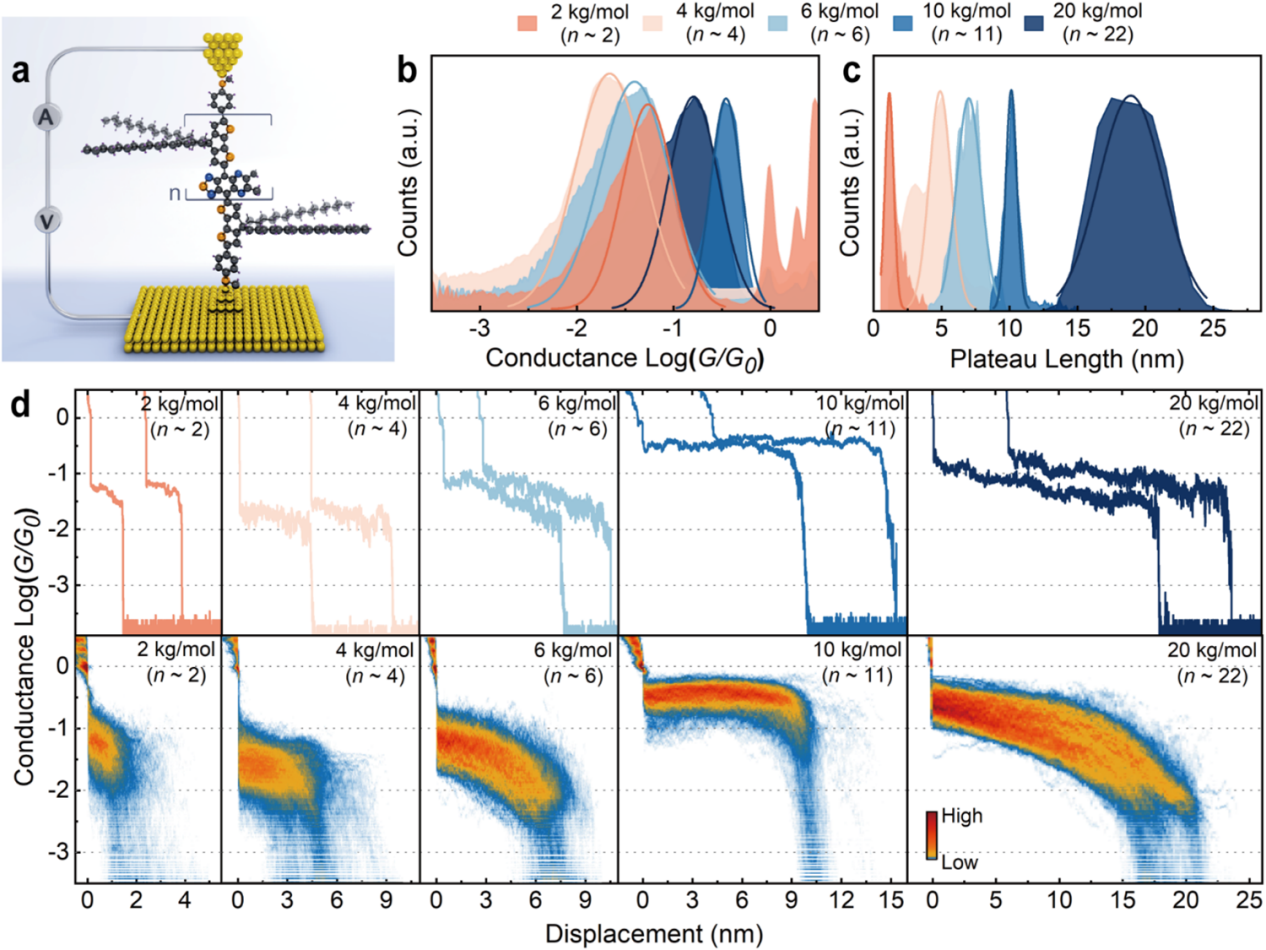
Conductance measurements of the open-shell donor–acceptor macromolecules. (a) Schematic of a single macromolecular junction formed in the STM-BJ setup. (b) 1D conductance histograms of the single macromolecules with molecular weight *M*_*n*_ of 2, 4, 6, 10, and 20 kg/mol measured under an applied bias of 200 mV in air under ambient condition. (c) Conductance plateau length histograms of the open-shell macromolecular series. (d) Representative conductance *vs* displacement traces (top) and 2D conductance histograms (bottom) for the corresponding open-shell macromolecule series (*M*_*n*_ of 2, 4, 6, 10, and 20 kg/mol). Around 3000 to 5000 traces were collected for each macromolecular sample and analyzed without data selection.

Notably, the observed transport behaviors in these macromolecules are of profound significance in multiple ways. First, the conductance of 10^–1.25^ *G*_0_ for the shortest macromolecule (*M*_*n*_ of ∼2 kg/mol (*n* ∼ 2)) is among the highest values reported for conjugated systems. As the molecular length increases, the conductance increases, yielding an inverse conductance attenuation of β = −0.08 nm^–1^ (Figure S16). This property, inaccessible with conventional solid-state electronic materials, leads to quasi-metallic transport in the two longest macromolecules (*M*_*n*_ of 10 and 20 kg/mol). Second, the highly efficient long-range transport occurs under a low applied bias in ambient conditions, which contrasts with previous studies, which required a high external bias^27,28^ and/or electrolyte^23^. Third, this quasi-metallic quantum transport is achieved across a molecular length surpassing 20 nm, extending the frontiers of molecular electronics into the tens of nanometers regime.

To understand the ultralong-range transport phenomena, we performed quantum transport calculations using the nonequilibrium Green’s function (NEGF) method combined with DFT at the (U)­B3LYP/6–31G** level of theory (see Supporting Information for details).^32,33^ The calculated transmission functions for the macromolecules with 4–7 repeat units (*n*) in their singlet electronic configuration are plotted in Figures 3a and S34. The triplet configuration is found to be less conductive and does not contribute to the zero-bias conductance (Figure S34e). As *n* increases, the bandgap rapidly narrows, accompanied by an evolution of the electronic structure arising from rapid configuration mixing (*i.e*., HOMO–LUMO mixing) as demonstrated by a decrease in the singlet–triplet gap (Δ*E*_ST_) and increase in diradical character (*y*) (Figure S24). The rapid bandgap reduction and adoption of a high-spin ground state at longer chain lengths with a thermally populated singlet state are consistent with absorption spectra, EPR, and previous reports.^29–31^ The well-defined single conductance peaks and the highly delocalized and spin-polarized FMOs are consistent with a highly coplanar geometry with pronounced quinoidal characteristics (Figure S25). Figure 3b depicts the α- and β-SOMOs of the singlet octamer (see Figures S26–S31 for *n* = 1 to 8). As the macromolecule becomes longer, there is a concomitant enhancement of spatial separation of progressively weaker interacting α- and β-spins and enhanced diradical character (*y* = 0.345 → 0.922 from *n* = 4 → 8; Table S3), resulting in edge-derived FMOs which become more localized toward the electrodes while retaining extensive spin-polarization throughout the conjugated backbone. Bond length alternation (BLA) analysis and nucleus-independent chemical shift (NICS) also reflect this increasing separation between the edge-derived diradical states (Figure S32 and Tables S4–S13). This topological feature of these strongly correlated diradicals promotes an ultrahigh transmission around *E*_F_, consistent with the increased conductance near 1 *G*_0_ observed in longer macromolecules, which is absent in other small molecular diradicals and topologically localized organic spin systems (*e.g.*, organic radicals, polycyclic aromatic hydrocarbons, magnetic edge states in graphene structures).^34,35^

**Figure 3:**
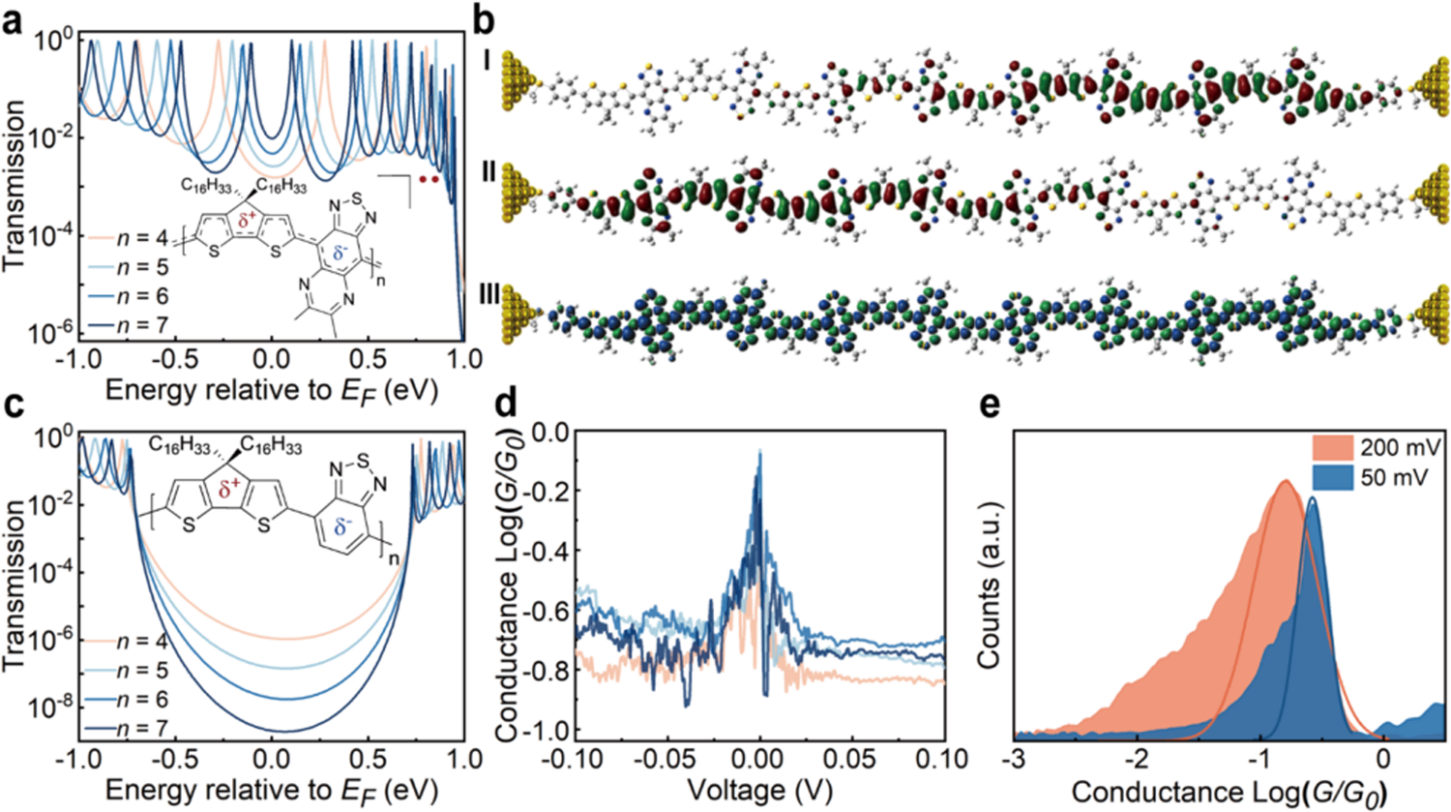
Correlation of theoretical and experimental transport measurements for open-shell donor–acceptor macromolecules and closed-shell counterpart. (a) Transmission function calculated using DFT-NEGF calculations for oligomers with 4, 5, 6, and 7 repeat units. The Fermi level for transport calculations was set in the middle of the transmission peaks associated with the FMOs. (b) DFT optimized geometric structure and frontier molecular orbitals of the *n* = 8 oligomer at the (U)­B3LYP/6–31G** level of theory: (I) α-SOMO, (II) β-SOMO, and (III) spin density distribution of the open-shell singlet. (c) Transmission functions for closed-shell counterpart with 4, 5, 6, and 7 repeat units. (d) Representative conductance *vs* applied bias (*G*–*V*) traces for open-shell macromolecules of 20 kg/mol (*n* ∼ 22) and (e) conductance histograms obtained at an applied bias of 50 and 200 mV. The solid lines represent Gaussian fits to the histograms.

As depicted in Figure 3a, the edge-state-derived resonances tend to merge at *E*_F_ as length increases, resulting in a conductance of approximately 1 *G*_0_ in the low-bias regime.^36^ Such behavior was experimentally captured for the long macromolecule of *M*_*n*_ = 20 kg/mol (*n* ∼ 22) using conductance *vs* applied bias (*G*–*V*) measurements. This phenomenon can be seen from the representative *G*–*V* traces yielding a pronounced peak rising toward 1 *G*_0_ at very low bias (<50 mV) (Figure 3d). To validate this result, we further conducted the static bias STM-BJ measurements at a lower bias of 50 mV, which resulted in a higher conductance compared to measurements at 200 mV (Figure 3e). This finding corroborates our hypothesis that the transmission for the long macromolecules occurs in the resonant regime, where FMOs are nearly degenerate. As a control, STM-BJ measurements of a closely related closed-shell analog poly­[4-(4,4-dihexadecyl-4*H*-cyclopenta­[2,1-*b*:3,4-*b*′]­dithiophen-2-yl)-*alt*-benzo­[*c*]­[1,2,5]­thiadiazole] displays no discernible conductance peak (Figure S17). The calculated transmission for the control macromolecule shows exponential decay with length (Figure 3c), consistent with previously reported closed-shell π-conjugated molecules and polymers.^20,27,37^

To manipulate the long-range transport in these macromolecules, we applied mechanical modulation, an effective approach to tailor charge transport through single molecules without gating.^38–40^ We carried out mechanically modulated STM-BJ measurements under an applied bias of 200 mV. In this measurement, the tip–substrate separation is repeatedly modulated by a distance of Δ*d* after the formation of a fully extended macromolecular junction (see Supporting Information for details). Figure 4a–c illustrate the results for *M*_*n*_ = 20 kg/mol (*n* ∼ 22) modulated with Δ*d* = 50 Å. A close correlation of conductance change with tip displacement was observed (Figures 4a and S21). Statistical analysis of hundreds of conductance traces reveals that mechanical compression of the junction results in a 4-fold enhancement in conductance, reaching 1 *G*_0_. This represents the first observation of unit transmission (or ballistic transport) in an ultralong single molecular wire in the low bias regime (Figure 4b,c). DFT calculations demonstrated that the macromolecule maintained its planar structure upon mechanical compression, leading to a negligible change in the transmission function. Therefore, the conductance enhancement can be primarily attributed to the stronger molecule-electrode coupling and broadening of the transmission resonances upon compression, owing to bonding of multiple sulfurs on the donor units with gold electrodes (Figure S35). Upon retraction, the total Au–S interaction is reduced and the transmission peaks become sharper leading to lower transmission at the Fermi level. The planar and rigid backbone emanating from intramolecular noncovalent S···N contacts and H-bonding interactions between the DA units of these macromolecular wires further motivated us to examine the junction mechanical stability (Figure S33). We conducted junction holding experiments for the *M*_*n*_ = 10 kg/mol and 20 kg/mol macromolecules (Figure S20). As depicted in Figure 4d, the representative trace for *M*_*n*_ = 10 kg/mol reveals an exceptionally long junction lifetime of >300 s, which contrasts with lifetimes of <10 s observed in previous STM-BJ measurements.^16^ This underpins the importance of optimized molecular design for creating robust molecular-scale devices.

**Figure 4:**
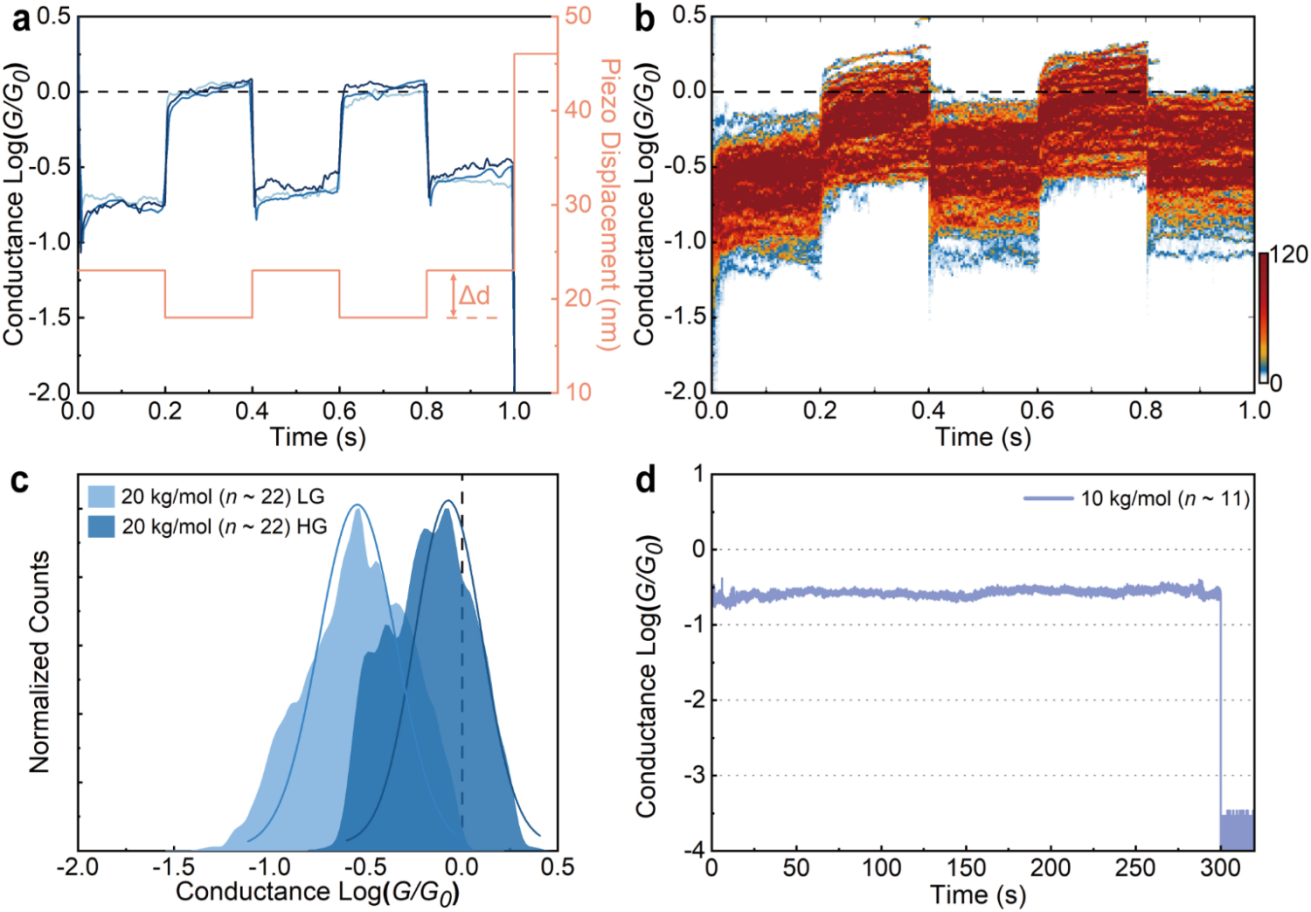
Mechanical modulation and junction holding measurements. (a) Representative conductance traces (blue) for the mechanically modulated junction of the 20 kg/mol open-shell macromolecule. Under a junction compression of Δ*d* = 50 Å (red), the conductance traces show reversible switching between high and low conductance states (HG and LG). The HG and LG states correspond to the compressed and fully extended junction configuration, respectively. The HG state reaches 1 *G*_0_, a signature of metallic ballistic quantum transport. (b) 2D conductance histograms of the mechanically modulated conductance traces for the 20 kg/mol macromolecule, and (c) corresponding 1D conductance histograms of the HG (dark blue) and LG (light blue) plateaus. Fitting the peaks with a Gaussian model gives 10^–0.62^ *G*_0_ and 10^–0.05^ *G*_0_ for the LG and HG states, respectively. (d) Conductance *vs* time trace for 10 kg/mol open-shell macromolecular junction.

We benchmarked the conductance of our macromolecules with other leading π-conjugated materials by plotting the measured single-molecule conductance against the calculated backbone length (Figure 5). Notably, the conductance values in this work significantly exceed other organic π-conjugated frameworks, including neutral and oxidized oligophenylenes,^23^ oligo­[*n*]­emeraldines^24^ which can be regarded as well-defined polyaniline fragments, neutral cumulenes,^25^ oligothiophenes,^20^ and diketopyrrolopyrrole oligomers,^41^ which are limited to <5 nm. Our values also greatly exceed those of graphene nanoribbons,^37^ and fused porphyrins.^27^ In addition, our macromolecular system exhibits superior performance in both chemical stability and transport length. As further detailed in Figure S22, our molecular design strategy simultaneously addresses several outstanding issues, including the inherently low conductance of organic materials, transmission decay with increasing backbone length, and instability issues associated with open-shell materials. This advancement, for the first time makes robust, ambient condition-compatible, low bias operatable, highly conductive organic materials viable for molecular electronics.

**Figure 5:**
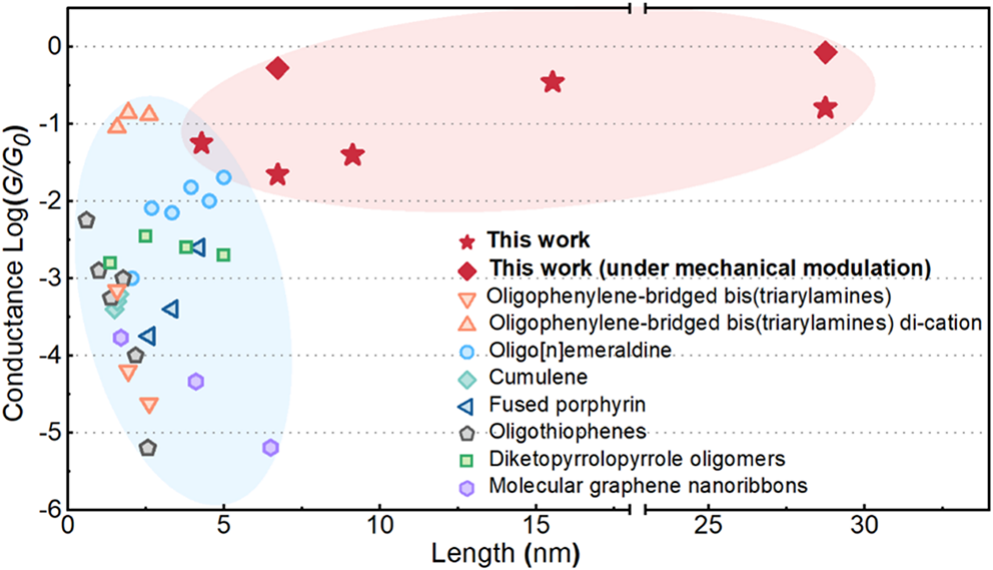
Comparison of experimental results of state-of-the-art π-conjugated molecules with the macromolecules reported in this work. For each work, the measured single-molecule conductance is plotted against the theoretically calculated molecular length. Molecules used in this comparison are oligophenylene-bridged bis­(triarylamines) in neutral and dication states,^23^ oligo­[*n*]­emeraldine,^24^ cumulene,^25^ fused porphyrin,^27^ oligothiophenes,^20^ diketopyrrolopyrrole oligomers,^41^ and molecular graphene nanoribbons.^37^

## Conclusions

By integrating emerging chemical design paradigms and quantum transport physics with single-molecule characterization, this work addresses a grand challenge in molecular electronics—achieving highly efficient long-range charge transport and establishing the basis for such functionality in organic materials. We present a model molecular wire system capable of facilitating resonant transport across several tens of nanometers in air at ambient conditions. This work provides direct experimental evidence that electronic wires made of ultralong organic molecules are capable of ballistic transport. In contrast to other open-shell systems, this macromolecular system is neutral, undoped, and stable in air, significantly enhancing its compatibility with modern solid-state nanotechnologies. Thus, neutral open-shell macromolecules are promising candidates for electronic wires, interconnects, and sensing components in molecular and nanoscale quantum devices. These materials open access to a variety of applications, including molecular electronics, organic (opto)­electronics, molecular spintronics, and quantum sensing, where long-range transport is central.

## Supplementary Material



## Notes

Synthetic methods, molecular weight control, macromolecular characterization using nuclear magnetic resonance (NMR) and matrix-assisted laser desorption ionization time-of-flight mass spectrometry (MALDI-TOF MS), optical characterization, variable temperature EPR measurements, general experimental methods for single-molecule conductance measurements, conductance-voltage characteristics, junction holding measurements, mechanical modulation measurements, additional data comparing the measured conductance of state-of-the-art π-conjugated molecules, cyclic voltammetry measurements, density functional theory (DFT) calculations; quantum transport calculations (PDF)
