# Supplementary material for: Long-Range Resonant Charge Transport through Open-Shell Donor–Acceptor Macromolecules
Source: J Am Chem Soc. 2025 May 1;147(24):20310–7. doi: 10.1021/jacs.4c18150 (PMC12186515; doi:10.1021/jacs.4c18150)
Supplement: Supplementary file 1 [file ja4c18150_si_001.pdf]

Supporting Information

Long-Range Resonant Charge Transport Through Open-Shell Donor-Acceptor Macromolecules

Shaocheng Shen[†1], *Mehrdad Shiri*[†2], *Paramasivam Mahalingam*[†3], *Chaolong Tang*[4], *Tyler Bills*[3], *Alexander J. Bushnell*[3], *Tanya A. Balandin*[3], *Leopoldo Mejía*[5], *Haixin Zhang*[2], *Bingqian Xu*[6], *Ignacio Franco*[*7,8], *Jason D. Azoulay*[*3], *Kun Wang*[*1,2]

[1]Department of Chemistry, University of Miami, Coral Gables, FL 33146, United States.

[2]Department of Physics, University of Miami, Coral Gables, FL 33146, United States.

[3]School of Chemistry and Biochemistry, School of Materials Science and Engineering, Center for Organic Photonics and Electronics, Georgia Institute of Technology, Atlanta, GA 30332, United States.

[4]Department of Physics and astronomy, Mississippi State University, Mississippi State, MS 39762, United States.

[5]Departamento de Física y Astronomía, Facultad de Ciencias Exactas, Universidad Andrés Bello, Santiago 837-0136, Chile.

[6]Single Molecule Study Laboratory, College of Engineering and Nanoscale Science and Engineering Center, University of Georgia, Athens, GA 30602, United States.

[7]Department of Chemistry, University of Rochester, Rochester, NY 14627, United States.

[8]Department of Physics, University of Rochester, Rochester, NY 14627, United States.

† These authors contributed equally to this work.

* Correspondence should be addressed to Ignacio Franco (ignacio.franco@rochester.edu), Jason D. Azoulay (jdazoulay@gatech.edu) and Kun Wang (kunwang@miami.edu).

Table of contents

1. Synthesis S3

1.1 Synthesis and end-functionalization of poly[4-(4,4-dihexadecyl-4*H*-cyclopenta[2,1-*b*:3,4-*b′*]dithiophen-2-yl)-*alt*-6,7-dimethyl-[1,2,5]thiadiazolo[3,4-*g*]quinoxaline] S3

2. General Procedure for Molecular Weight Control S4

2.1 Polymer Target: M_n = 20 kg/mol S4

2.2 Polymer Target: M_n = 10 kg/mol S4

2.3 Polymer Target: M_n = 6 kg/mol S5

2.4 Polymer Target: M_n = 4 kg/mol S5

2.5 Polymer Target: M_n = 2 kg/mol S5

3. Materials Characterization S10

3.1 UV-Vis-NIR spectroscopy S10

3.2 Electron Paramagnetic Resonance Spectroscopy S14

4. Electrical Characterizations S15

4.1 Conductance measurements S15

4.2 Conductance-Voltage (*G*-*V*) measurements S19

4.3 Junction holding measurements S20

4.4 Mechanical modulation S21

4.5 Cyclic voltammetry S25

5. Theoretical Calculations S26

5.1 DFT Calculations. S26

5.2 Transmission calculations. S43

6. References S46

1. Synthesis

All manipulations of air- and moisture-sensitive compounds were performed under an inert atmosphere using standard glovebox and Schlenk techniques. Reagents, unless otherwise specified, were purchased from Fischer Scientific and used without further purification. Xylenes was dried over 4 Å molecular sieves prior to use. 1,1,2,2-tetrachloroethane-d_2 was purchased from Cambridge Isotope Labs and dried over 4 Å molecular sieves prior to use. Tetrakis(triphenylphosphine)palladium(0) was purchased from Strem Chemicals and used as received. 4,4-Dihexadecyl-4*H*-cyclopenta[2,1-*b*:3,4-*b′*]dithiophene,[1] (4,4-dihexadecyl-4*H*-cyclopenta[2,1-*b*:3,4-*b′*]dithiophene-2,6-diyl)bis(trimethylstannane) (**M1**),[2] and 4,9-dibromo-6,7-dimethyl-[1,2,5]thiadiazolo[3,4-*g*]quinoxaline (**M2**)[3] were prepared according to literature procedures. ^{1}H NMR spectra were collected on a Bruker Avance III 400 MHz spectrometer and chemical shifts, δ (ppm), were referenced to the residual solvent impurity peak of the given solvent. Data reported as: s = singlet, d = doublet, t = triplet, m = multiplet, br = broad; coupling constants (J) are given in Hz. Microwave-assisted reactions were performed in a CEM Discover 2.0 microwave reactor. The number average molecular weight (M_n) and dispersity ($Đ$) were determined by gel permeation chromatography (GPC) relative to polystyrene standards at 160 °C in 1,2,4-trichlorobenzene using a Tosoh EcoSEC HT GPC system configured with TSKgel GMH_{HR}-H(S) HT2 columns in series. Polymer samples were prepared at a concentration of 1 to 2 mg/ml in 1,2,4-trichlorobenzene at 160 °C.

1.1 Synthesis and end-functionalization of poly[4-(4,4-dihexadecyl-4*H*-cyclopenta[2,1-*b*:3,4-*b′*]dithiophen-2-yl)-*alt*-6,7-dimethyl-[1,2,5]thiadiazolo[3,4-*g*]quinoxaline]

A microwave tube was loaded with **M1** (100 mg, 0.105 mmol, 1 equiv.) and **M2** (37.3 mg, 0.100 mmol, 0.95 equiv.). The vessel was transferred to a nitrogen-filled glovebox where 480 μL of a $Pd(PPh_3)_4$/xylenes stock solution (3.5 mol%) was added. The tube was sealed and then heated, while stirring, in a microwave reactor using the following sequence: 120 °C for 5 min, 140 °C for 5 min, and 170 °C for 30 min. Subsequently, 1 mL of a $Pd(PPh_3)_4$/xylenes stock solution (1 mol%) and 10 equivalents of 4-bromothioanisole (213 mg, 1.050 mmol) were added. This mixture was stirred for 12 hours at 100 °C to functionalize the polymer chain ends. After this time, the reaction was cooled, and the mixture was precipitated into methanol and collected via filtration. The residual solid was loaded into an extraction thimble and successively washed with methanol (2

hours), acetone (2 hours), hexanes (16 hours), and then acetone (2 hours). The polymer was dried *in vacuo* to give 77.2 mg (91%) of a black solid. ^{1}H NMR (400 MHz, 1,1,2,2-tetrachloroethane-d_2, 398 K): δ 9.19 (br, 2H), 7.31−7.09 (br, 4H), 3.03 (br, 6H), 2.23 (br, 4H), 2.05 (br, 4H), 1.29 (br, 52H), 0.95 (s, 6H). Absorption: λ_{max} (thin film) = 1576 nm. Spectra were consistent with previous reports.[4]

2. General Procedure for Molecular Weight Control

The molecular weight was varied by modifying the stoichiometric ratio (*r*) of the reaction with **M1** in excess relative to **M2** in accordance with Carothers' equation (Eq. S1).

$$M_n = M_0 \left[\frac{1+r}{1+r-2rp}\right] \qquad \text{(Eq. S1)}$$

Here, M_n represents the target number average molecular weight, M_0 represents the average monomer weight, and p is the extent of conversion. Using the conditions detailed above, stoichiometric ratios of r = 0.50 (**M2** = 19.6 mg, 0.052 mmol), r = 0.70 (**M2** = 27.5 mg, 0.073 mmol), r = 0.80 (**M2** = 31.4 mg, 0.083 mmol), r = 0.90 (**M2** = 35.3 mg, 0.089 mmol), and r = 0.95 (**M2** = 37.3 mg, 0.094 mmol), were used to target an M_n ~ 2, 4, 6, 10, and 20 kg/mol, respectively. After polymerization and end-functionalization, the reaction mixtures were cooled, precipitated into methanol, and purified by Soxhlet extraction according to the optimized procedures below.

2.1 Polymer Target: M_n = 20 kg/mol

The polymer was purified by Soxhlet extraction with methanol (2 hours), acetone (2 hours), hexanes (16 hours), and then acetone (2 hours). The polymer was dried *in vacuo* to give 77.2 mg (91%) of a black solid. GPC analysis (160 °C, TCB): M_n = 17.3 kg/mol and $Đ$ =1.11. ^{1}H NMR (400 MHz, 1,1,2,2-tetrachloroethane-d_2, 398 K): δ 9.19 (br, 2H), 7.31−7.09 (br, 4H), 3.03 (br, 6H), 2.23 (br, 4H), 2.05 (br, 4H), 1.29 (br, 52H), 0.95 (s, 6H). Absorption: λ_{max} (thin film) = 1576 nm.

2.2 Polymer Target: M_n = 10 kg/mol

The polymer was purified by Soxhlet extraction residual with methanol (2 hours), acetone (2 hours), hexanes (16 hours), and then acetone (2 hours). The polymer was dried *in vacuo* to give

71.3 mg (91%) of a black solid. GPC analysis (160 °C, TCB): M_n = 8.4 kg/mol and $Đ$ = 1.13. ^{1}H NMR (400 MHz, 1,1,2,2-tetrachloroethane-d_2, 398 K): δ 9.20 (br, 2H), 7.30−7.09 (br, 4H), 3.03 (br, 6H), 2.59 (s, 3H), 2.32 (br, 4H), 2.08 (br, 4H), 1.29 (br, 52H), 0.93 (br, 6H). Absorption: λ_{max} (thin film) = 1544 nm.

2.3 Polymer Target: M_n = 6 kg/mol

The polymer was purified by Soxhlet extraction residual with methanol (2 hours), acetone (2 hours), a 7:3 mixture of hexanes and acetone (16 hours), and then acetone (2 hours). The polymer was dried *in vacuo* to give 61.1 mg (82%) of a black solid. GPC analysis (160 °C, TCB): M_n = 4.93 kg/mol and $Đ$ = 1.37. ^{1}H NMR (400 MHz, 1,1,2,2-tetrachloroethane-d_2, 398 K): δ 9.18 (br, 2H), 7.63−7.39 (br, 4H), 3.02 (br, 6H), 2.59 (s, 3H), 2.23 (br, 4H), 2.08 (br, 4H) 1.29 (br, 52H), 0.95 (br, 6H). Absorption: λ_{max} (thin film) = 1525 nm.

2.4 Polymer Target: M_n = 4 kg/mol

The polymer was purified by Soxhlet extraction residual with methanol (2 hours), acetone (2 hours), a 1:1 mixture of hexanes and acetone (16 hours), and then acetone (2 hours). The polymer was dried *in vacuo* to give 52.4 mg (84%) of a black solid. GPC analysis (160 °C, TCB): M_n = 3.41 kg/mol and $Đ$ = 1.17. ^{1}H NMR (400 MHz, 1,1,2,2-tetrachloroethane-d_2, 398 K): δ 9.19 (br, 2H), 7.62−7.39 (br, 4H), 3.02 (br, 6H), 2.59 (s, 3H), 2.23 (br, 4H), 2.09 (br, 4H), 1.29 (br, 52H), 0.95 (br, 6H). Absorption: λ_{max} (thin film) = 1293 nm.

2.5 Polymer Target: M_n = 2 kg/mol

The polymer was purified by Soxhlet extraction residual with methanol (2 hours), acetone (2 hours), a 2:8 mixture of hexanes and acetone (16 hours), and then acetone (2 hours). The polymer was dried *in vacuo* to give 29.5 mg (67%) of a black solid. GPC analysis (160 °C, TCB): M_n = 1.54 kg/mol and $Đ$ = 1.69. ^{1}H NMR (400 MHz, 1,1,2,2-tetrachloroethane-d_2, 398 K): δ 9.17 (br, 2H), 7.62−7.39 (br, 4H), 2.97 (br, 6H), 2.59 (s, 3H), 2.21 (br, 4H), 2.08 (br, 4H), 1.29 (br, 52H), 0.95 (br, 6H). Absorption: λ_{max} (thin film) = 1132 nm.

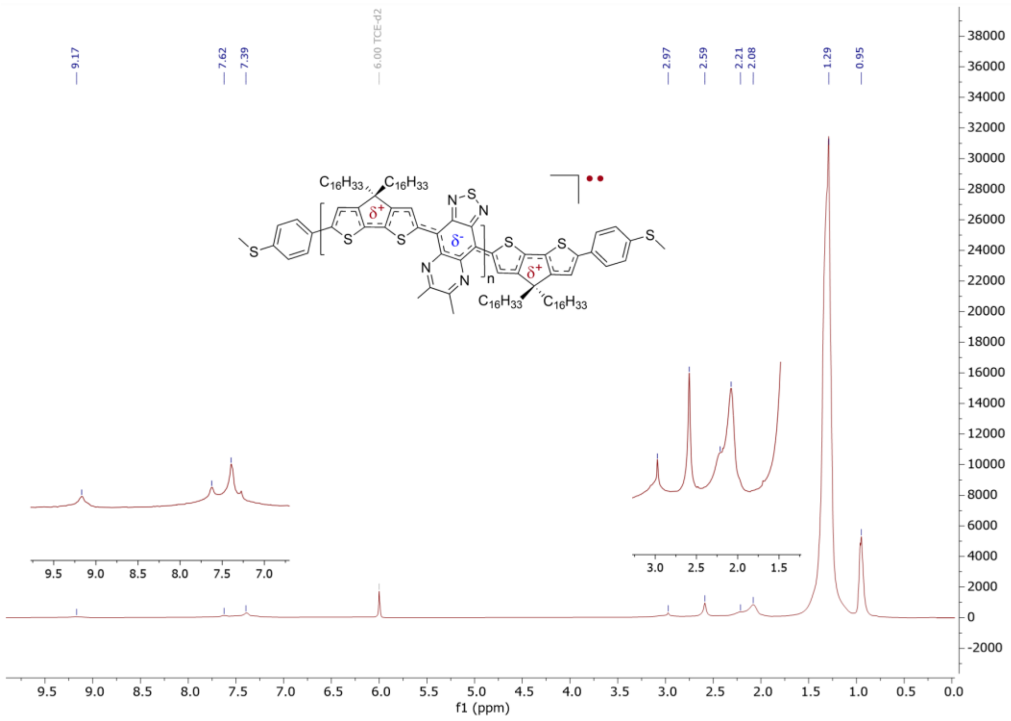

Figure S1. ^{1}H NMR spectra of M_n = 2 kg/mol (r = 0.50) open-shell macromolecule (400 MHz, 1,1,2,2-Tetrachloroethane-d_2, 398 K).

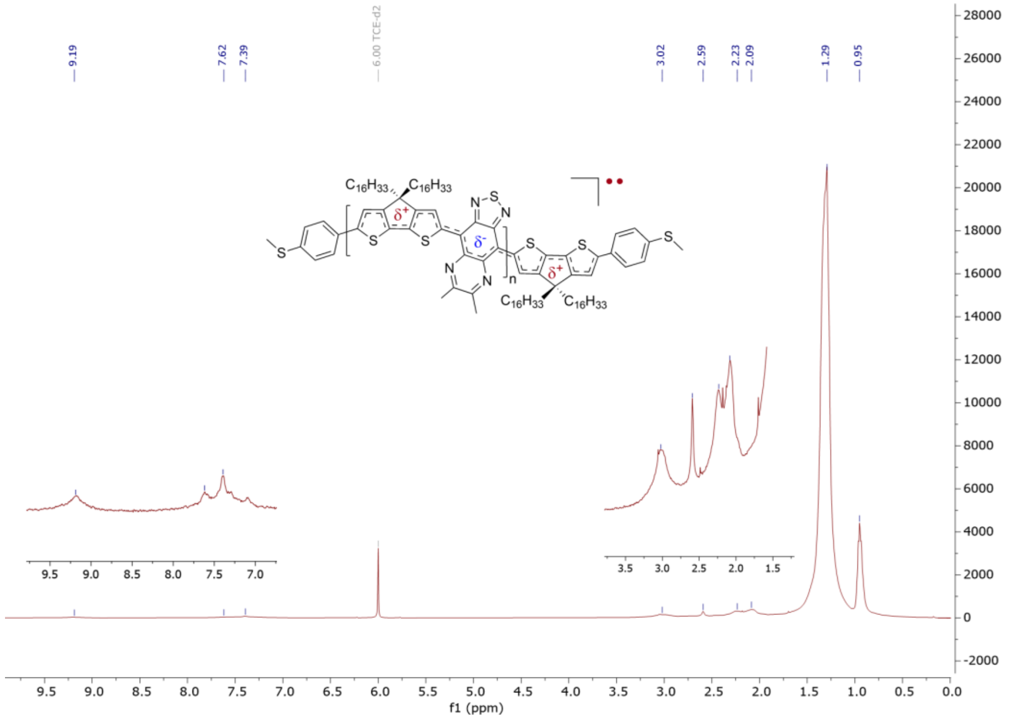

Figure S2. ^{1}H NMR spectra of M_n = 4 kg/mol (r = 0.70) open-shell macromolecule (400 MHz, 1,1,2,2-Tetrachloroethane-d_2, 398 K).

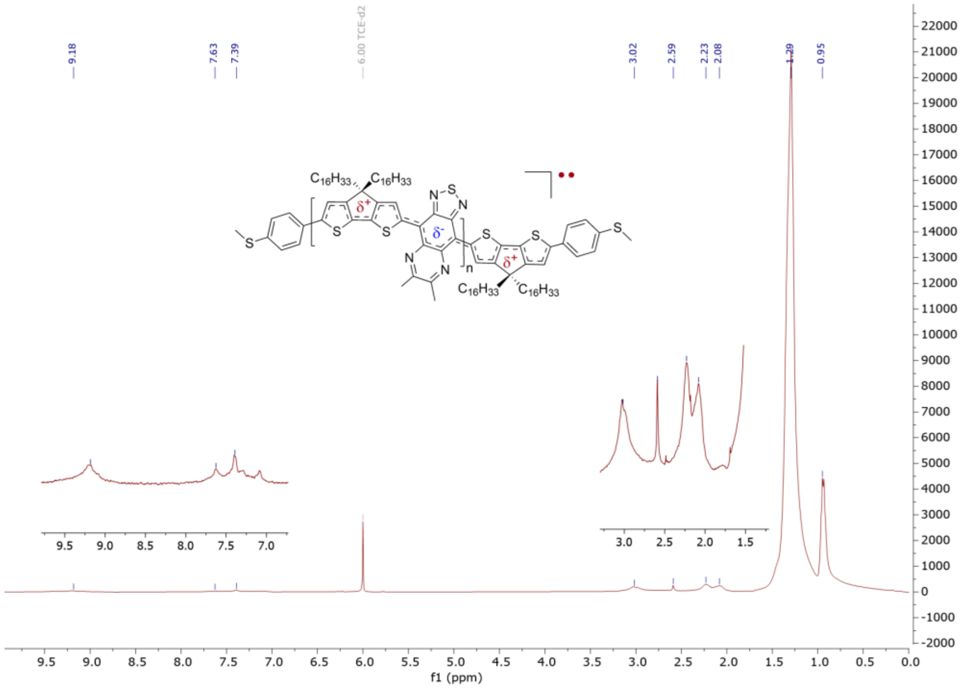

Figure S3. ^{1}H NMR spectra of M_n = 6 kg/mol (r = 0.80) open-shell macromolecule (400 MHz, 1,1,2,2-Tetrachloroethane-d_2, 398 K).

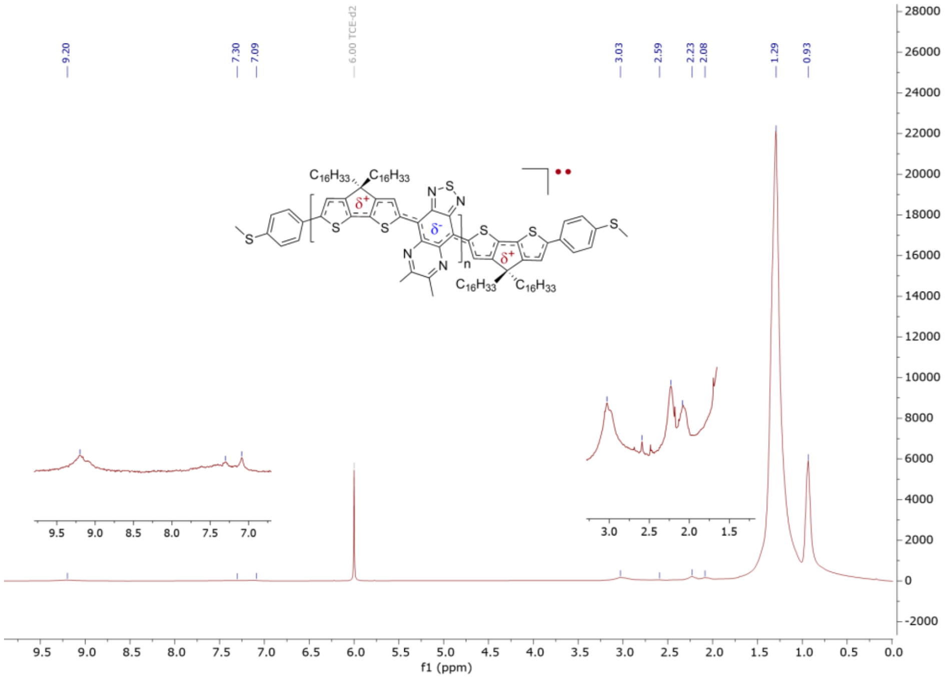

Figure S4. ^{1}H NMR spectra of M_n = 10 kg/mol (r = 0.90) open-shell macromolecule (400 MHz, 1,1,2,2-Tetrachloroethane-d_2, 398 K).

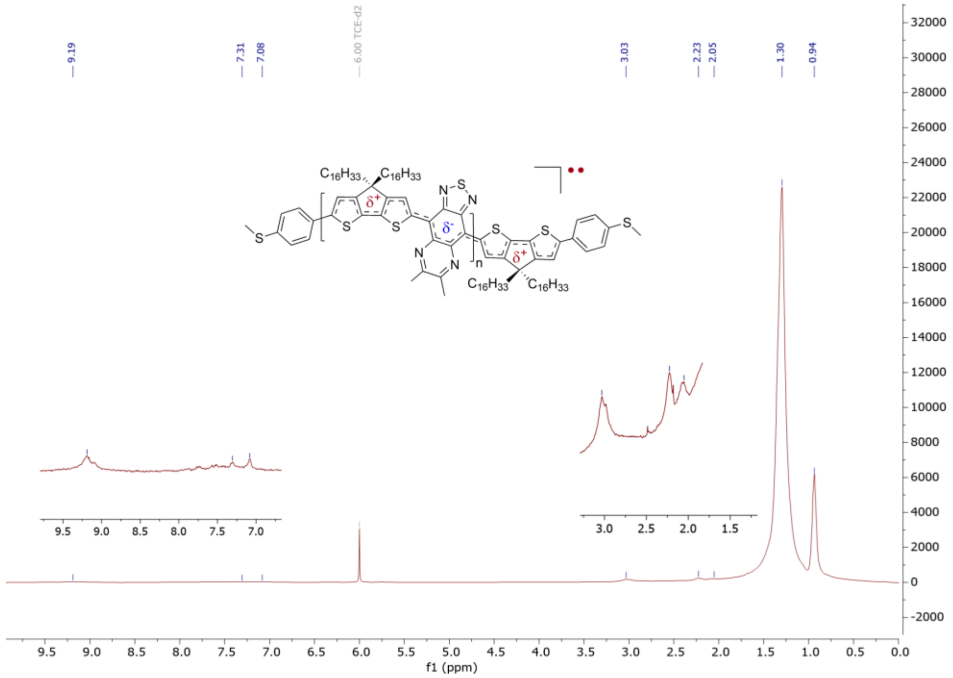

Figure S5. ^{1}H NMR spectra of M_n = 20 kg/mol (r = 0.95) open-shell macromolecule (400 MHz, 1,1,2,2-Tetrachloroethane-d_2, 398 K).

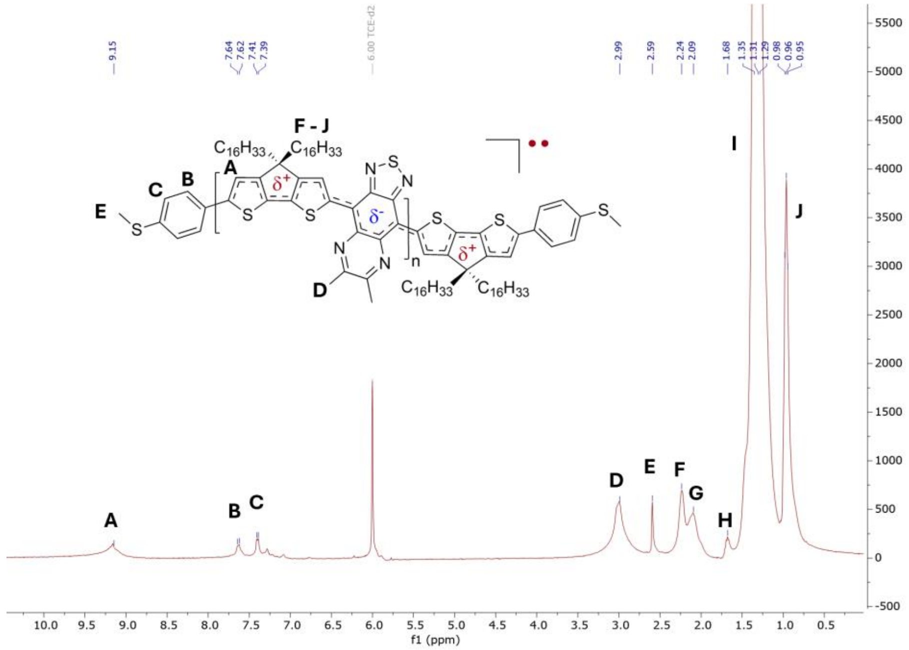

Figure S6. Proton assigned ^{1}H NMR spectra of M_n = 4 kg/mol (r = 0.70) open-shell macromolecule in optimized NMR conditions (400 MHz, 1,1,2,2-Tetrachloroethane-d_2, 388 K).

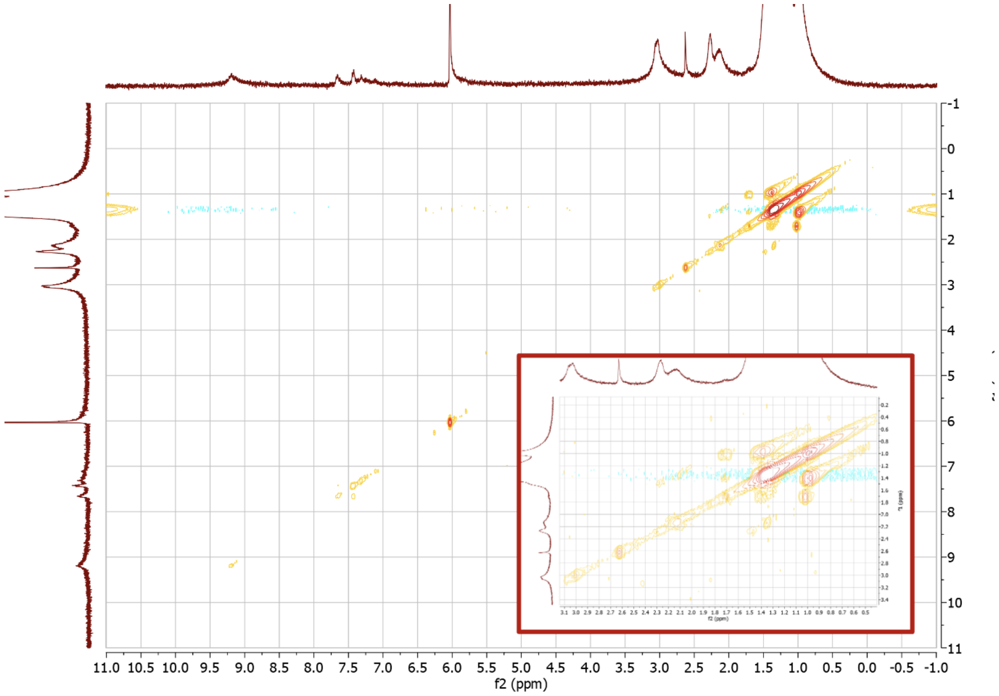

Figure S7. 2-D ^{1}H correlation spectroscopy (COSY) spectra of M_n = 4 kg/mol (r = 0.70) open-shell macromolecule (400 MHz, 1,1,2,2-Tetrachloroethane-d_2, 388 K). Correlation peaks are consistent with the ^{1}H assignments in Figure S6.

Matrix-assisted laser desorption ionization time of flight mass spectrometry (MALDI-TOF MS) of a polymerization reaction mixture was taken with a Bruker RapiFlex MALDI-TOF mass spectrometer. The matrix was prepared with a 10 mg/mL solution of *trans*-2-[3-(4-*tert*-Butylphenyl)-2-methyl-2-propenylidene]malononitrile (DCTB) in DCM. 5 mg of the sample was dissolved in 200 µL of DCM, and 2 µL of the resulting solution was mixed with 20 µL of the matrix. 0.5 µL of the final mixture was spotted on a stainless-steel target plate and analyzed in the linear mode. The results are consistent with the polymer structure where the thioanisole end-capped n = 1 unit has a theoretical mass of 1710-1716 Da (depending on isotopic content) and a peak with a m/z value of 1712.71 is observed. Each subsequent cluster of peaks is evenly spaced by ~838.5 Da (the repeat unit mass) corresponding to n = 2 – 6 polymer chains.

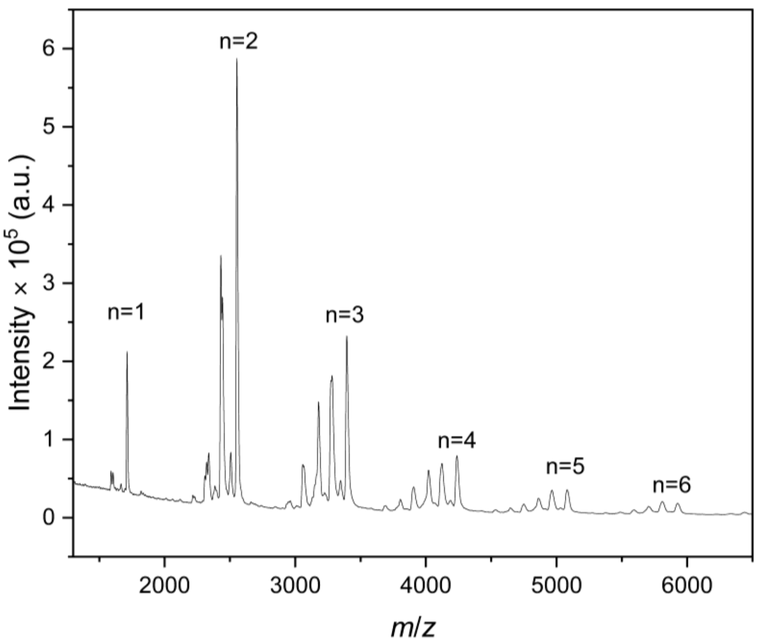

Figure S8. Matrix-assisted laser desorption ionization time of flight mass spectrometry (MALDI-TOF MS) of a polymerization reaction mixture.

3. Materials Characterization

3.1 UV-Vis-NIR spectroscopy

UV–vis–NIR and Fourier transform infrared (FTIR) spectra were recorded from 0.20 to 3.30 μm and from 1.3 to 25.0 μm using a Cary 5000 UV–vis–NIR spectrophotometer and Shimadzu IRAffinity-1 FTIR spectrometer respectively. Thin films were prepared by spin coating a chlorobenzene solution (10 mg/mL) onto quartz and KBr substrates at 1000 rpm.

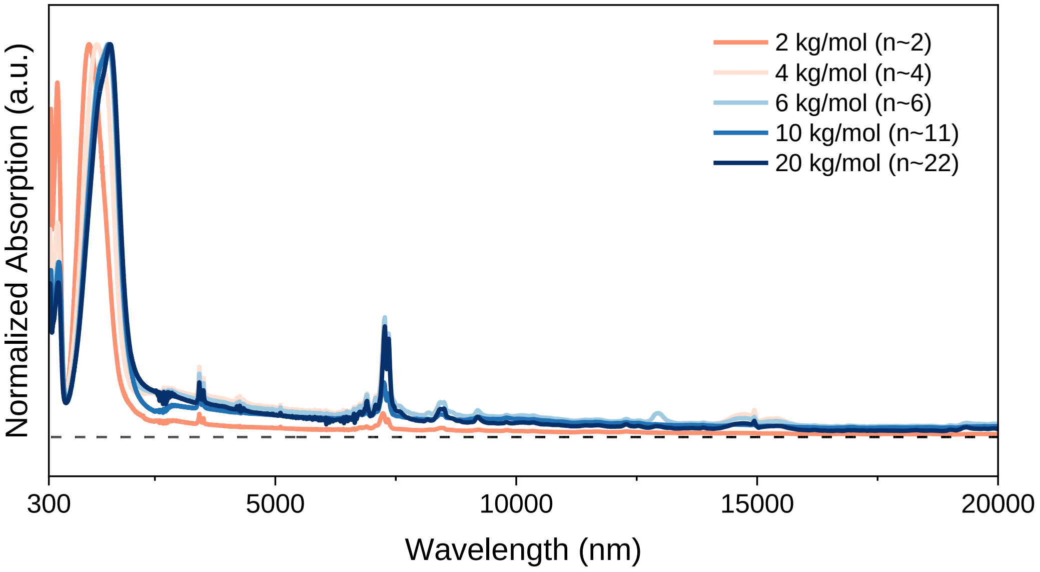

Figure S9. Absorption spectra of thin films cast from chlorobenzene onto quartz and KBr substrates.

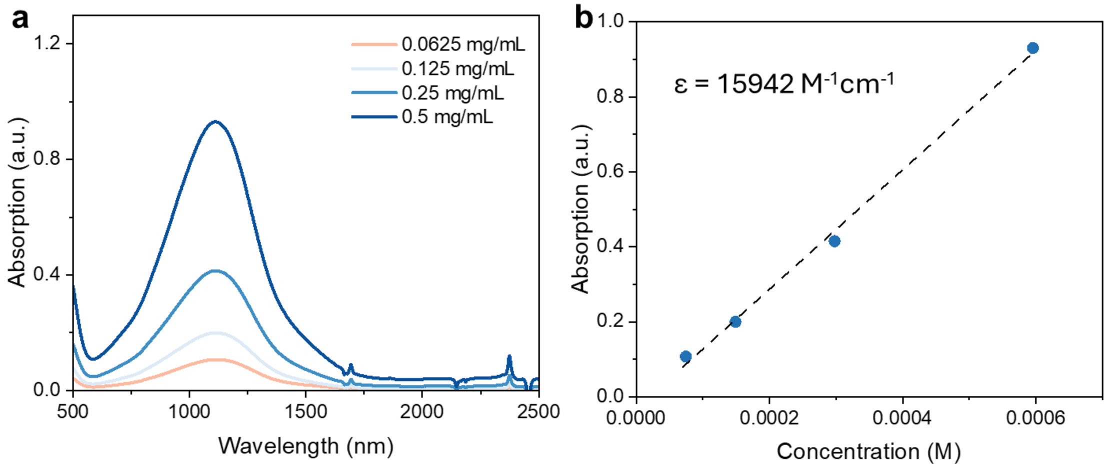

Figure S10. a) Solution absorption spectra of the 2 kg/mol polymer serially diluted in chlorobenzene. b) The molar absorptivity was determined via a linear fit according to the Beer-Lambert law.

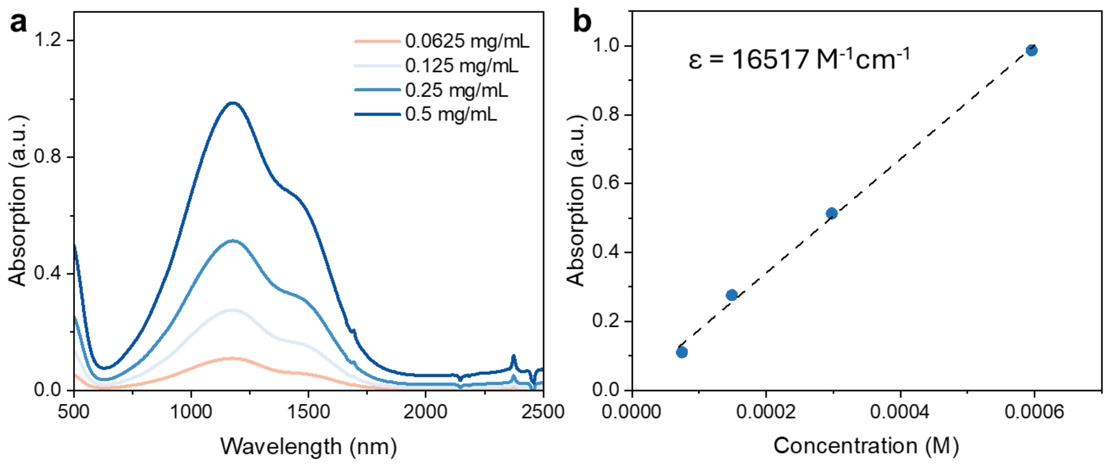

Figure S11. a) Solution absorption spectra of the 4 kg/mol polymer serially diluted in chlorobenzene. b) The molar absorptivity was determined via a linear fit according to the Beer-Lambert law.

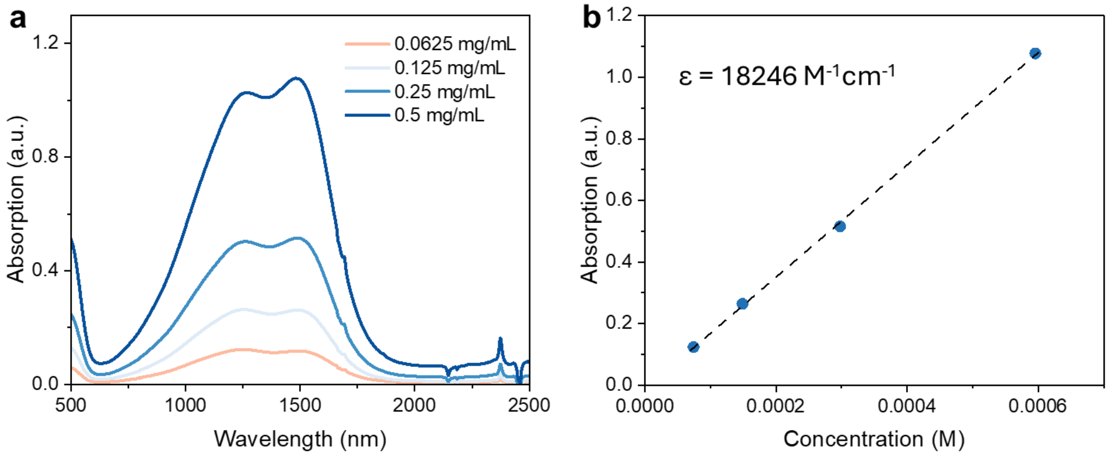

Figure S12. a) Solution absorption spectra of the 6 kg/mol polymer serially diluted in chlorobenzene. b) The molar absorptivity was determined via a linear fit according to the Beer-Lambert law.

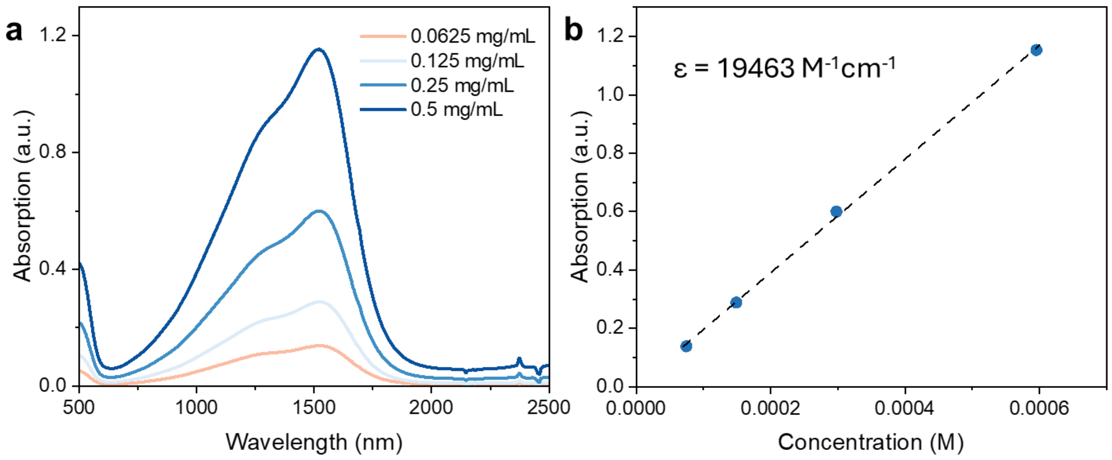

Figure S13. a) Solution absorption spectra of the 10 kg/mol polymer serially diluted in chlorobenzene. b) The molar absorptivity was determined via a linear fit according to the Beer-Lambert law.

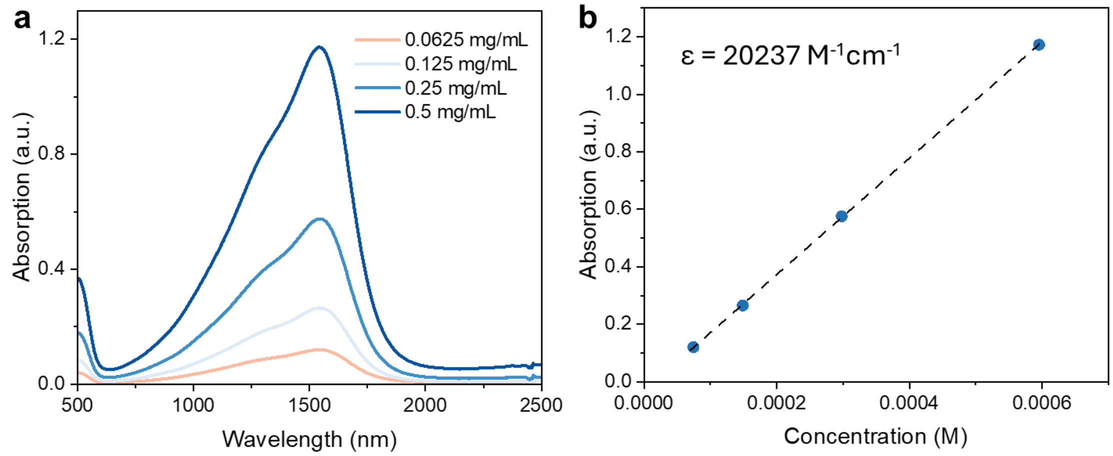

Figure S14. a) Solution absorption spectra of the 20 kg/mol polymer serially diluted in chlorobenzene. b) The molar absorptivity was determined via a linear fit according to the Beer-Lambert law.

Table S1. Summarized thin film and solution state absorption data for the polymer series.

M_n	Film E_{max} (eV)	Film λ_{max} (nm)	Solution E_{max} (eV)	Solution λ_{max} (nm)	Molar Absorptivity ($M^{-1}cm^{-1}$)
2	1.095	1132	1.113	1114	15942
4	0.959	1293	1.049	1182	16517
6	0.813	1525	0.836	1484	18246
10	0.803	1544	0.816	1520	19463
20	0.787	1576	0.803	1545	20237

3.2 Electron Paramagnetic Resonance Spectroscopy

Room temperature continuous-wave EPR spectra were recorded on a Bruker E500 EPR spectrometer operating in the X-band. Solid-state samples were loaded into 4 mm quartz tubes and evacuated for 12 hours before sealing under vacuum.

Variable temperature EPR spectra were collected on solution-state samples that were prepared in anhydrous chlorobenzene (0.4 mg/mL), and the 4 mm quartz tubes were flame sealed under vacuum. The data were collected from 25 to 5 K for the chlorobenzene glass. The EPR signal intensity from 20 to 5 K was utilized to extract the singlet–triplet energy splitting (ΔE_{ST}) through fitting the data to the Bleaney–Bowers equation:

$$I_{\text{EPR}} = \frac{C}{T}\frac{3e^{-2J/k_{\text{B}}T}}{1+3e^{-2J/k_{\text{B}}T}} \qquad \text{(Eq. S2)}$$

where C is a constant, J is the intramolecular exchange coupling constant, and 2J is ΔE_{ST}. A dilute solution (0.4 mg/mL) was used to elucidate the single-chain behavior. The fit parameters, J = 1.14 cm^{-1} and $\Delta E_{ST} = 6.48 \times 10^{-3}$ kcal/mol were obtained.

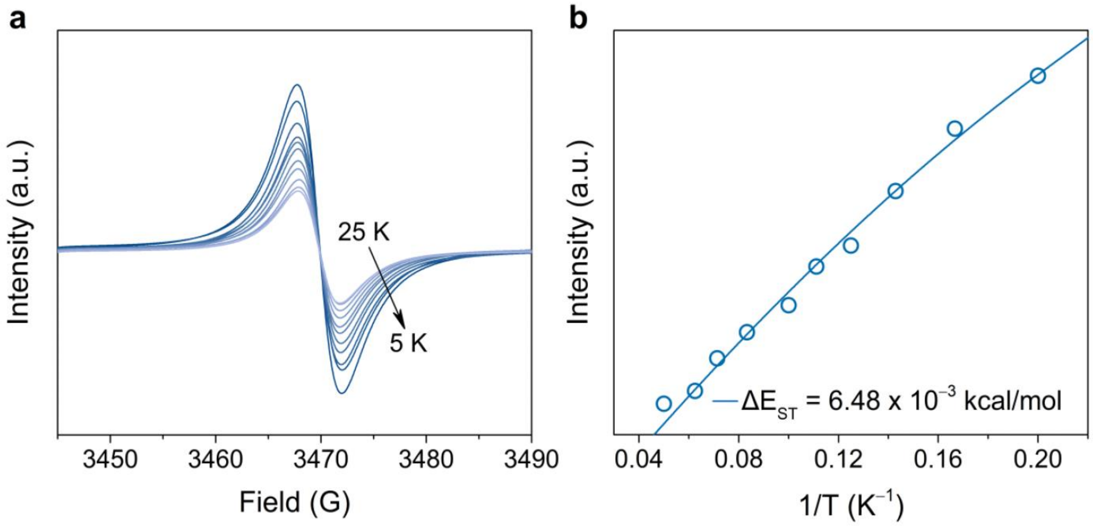

Figure S15. a) EPR spectra of dilute macromolecular solution in anhydrous chlorobenzene (0.4 mg/mL) measured from 25 to 5 K and b) temperature-dependent fit to the Bleaney–Bowers equation with ΔE_{ST} of 6.48×10^{-3} kcal/mol.

4. Electrical Characterizations

4.1 Conductance measurements

We carried out the single-molecule conductance measurements of the synthesized macromolecules using the scanning tunneling microscope break-junction technique, as detailed previously.[5] Specifically, an electrochemically etched gold (Au) wire and a freshly annealed gold (111) surface on mica were used as the STM tip and substrate, respectively. The macromolecules with varying molecular weights (M_n of 2, 4, 6, 10, and 20 kg/mol) were dissolved in chloroform with 0.01mM-0.05 mM concentration and used as macromolecular solutions. The gold substrate was immersed in the macromolecular solution for incubation of 2 ~ 3 hours, followed by thorough rinsing with chloroform to remove excessive macromolecules. The macromolecule-coated gold substrate was then dried with nitrogen gas for subsequent electrical measurements. In the STM-BJ measurements, we used a custom-designed LabVIEW program to control the tip-substrate separation with sub-angstrom-level precision. Briefly, the tip was first driven to make contact with the macromolecule-coated gold substrate and then retract from the surface at a speed of 10 nm/s. Upon rupture of the gold atomic contact, a macromolecule could bridge the tip-substrate gap to form an Au-macromolecule-Au junction, as evidenced by a plateau in the conductance *vs.*

displacement trace. Under a static applied voltage V, the electrical conductance (G) (current (I) /voltage (V)) of the junction as a function of tip displacement was continuously recorded at a 13 kHz acquisition rate. Data collected from the tip retraction process, as shown in the conductance *vs.* displacement traces, was used to analyze the conductance of the tested macromolecules. We collected 3,000 to 5,000 traces under V = 200 mV for each macromolecule and used them to construct the corresponding 1D and 2D conductance histograms. All measurements were conducted in air under ambient conditions.

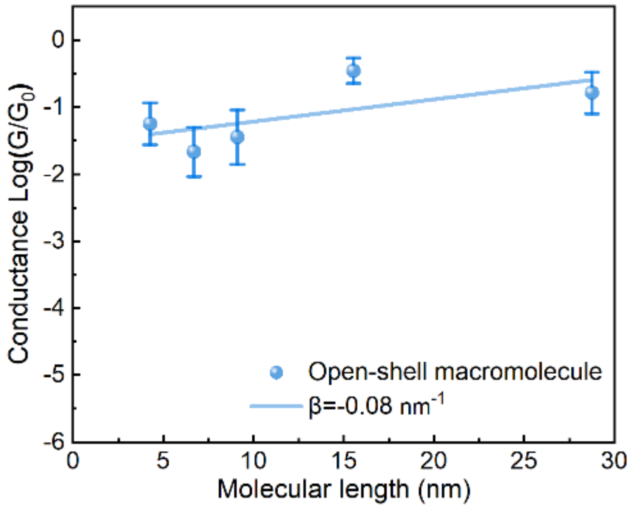

Figure S16. Experimentally measured conductance values for open-shell macromolecules with M_n of 2, 4, 6, 10, and 20 kg/mol as a function of calculated length. The slope of the fitting line was used to find the β value. The error bars correspond to the full width at half maximum (FWHM) of the gaussian fitting peaks in conductance histograms.

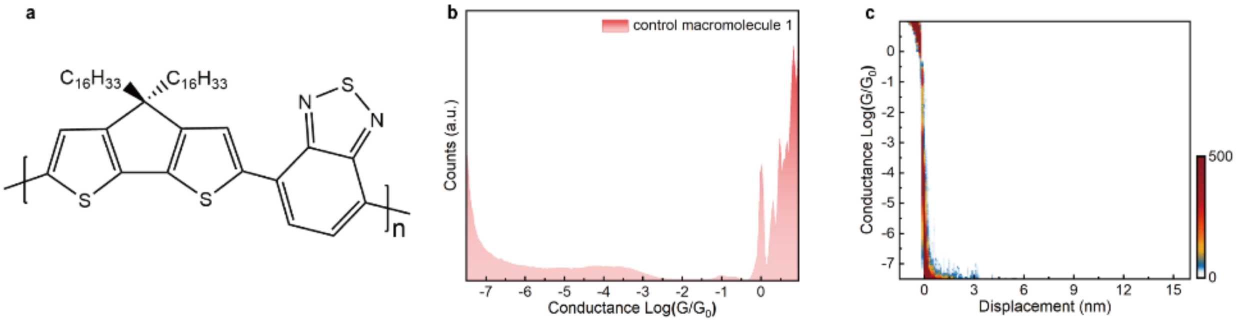

Figure S17. a) Molecular structure of the closed-shell control macromolecule with M_n of 10 kg/mol (n ~ 12). b) 1D and c) 2D conductance histograms of the closed-shell control macromolecule with M_n of 10 kg/mol (n ~ 12) measured under 200 mV applied bias in air under ambient conditions. A logarithmic current amplifier was used to check molecular conductance

features in a broad current range. Both 1D and 2D histograms show that the closed-shell control macromolecules are nonconducting, as the conductance is below the noise floor of our STM-BJ instrument (10^{-7} G_0).

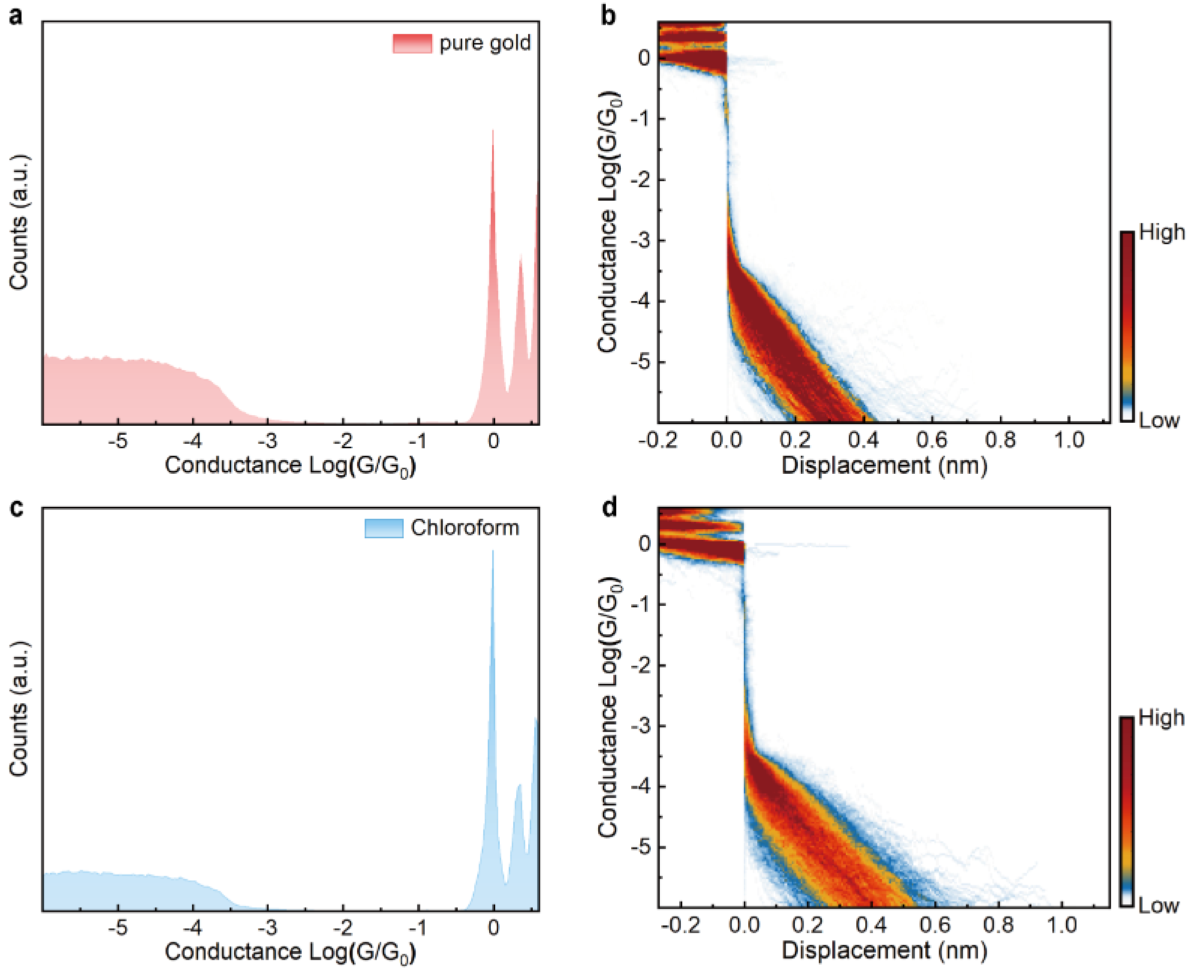

Figure S18. a) 1D and b) 2D histograms of conductance data obtained from STM-BJ measurement on pristine gold as the control experiment. c) 1D and d) 2D histograms of conductance data obtained from STM-BJ measurement on chloroform deposited on the sample and dried out as the control experiment.

Table S2. Molecular weight, theoretical and experimental length, and conductance of single conductive open-shell macromolecule series and their closed shell counterpart as control macromolecule. The control macromolecule has the acceptor unit modified.

**Molecule**	**Theoretical Length (nm)**	**Experimental Length (nm)**	**Theoretical Conductance (log(*G*/*G*₀))**	**Experimental Conductance (log(*G*/*G*₀))**	**Molecular Weight (g/mol)**
Open-shell macromolecule (*n*=1)	3.061		-2.5624		1710.86
Open-shell macromolecule (*n*=2)	4.2843	1.66 (2 kg/mol)	-2.7881	-1.254	2550.52
Open-shell macromolecule (*n*=3)	5.5090		-2.7881		3390.18
Open-shell macromolecule (*n*=4)	6.7144	5.40 (4 kg/mol)	-2.6325	-1.660	4229.84
Open-shell macromolecule (*n*=5)	7.9215		-2.1057		5069.5
Open-shell macromolecule (*n*=6)	9.1224	7.50 (6 kg/mol)	-1.5029	-1.406	5909.16
Open-shell macromolecule (*n*=7)	10.3461		-1.0778		6748.82
Open-shell macromolecule (*n*=11)	~15.53	10.61 (10 kg/mol)		-0.460	10107.46
Open-Shell macromolecule (*n*=22)	~28.7503	19.42 (20 kg/mol)		-0.795	19343.72
Closed-Shell control macromolecule (*n*=4)	6.7011		-5.9225		
Closed-Shell control macromolecule (*n*=5)	7.9111		-6.7856		
Closed-Shell control macromolecule (*n*=6)	9.1128		-7.6804		
Closed-Shell control macromolecule (*n*=7)	10.3333		-8.6413		
Closed-Shell control macromolecule (*n*=12)	~17.087			Below noise floor*	~10000

* Our instrument noise floor is 10^{-7} G_0.

4.2 Conductance-Voltage (*G-V*) measurements

We characterized the conductance-voltage (*G-V*) characteristics of the 20 kg/mol (*n* ~ 22) macromolecular junction by monitoring the junction current (*I*) under a continuous sweep of applied bias *V*, as described previously.[6] Specifically, the STM tip was retracted from the gold surface to form a single-molecule junction under a static bias of 200 mV. In this measurement, a fixed tip retraction distance was used to purposely form a molecular junction in which the entire molecular backbone is fully extended. This distance, here 20 nm, was determined by the most probable plateau length extracted from the 2D conductance *vs.* displacement histogram of 20 kg/mol (*n* ~ 22) macromolecule. After the formation of a fully extended molecular junction, we held the junction for 1 second and then swept the applied bias continuously from -0.1V to +0.1V. For each cycle, the collected current (*I*) was converted to conductance (*G*) by dividing the voltage (*V*) to construct the *G-V* curves.

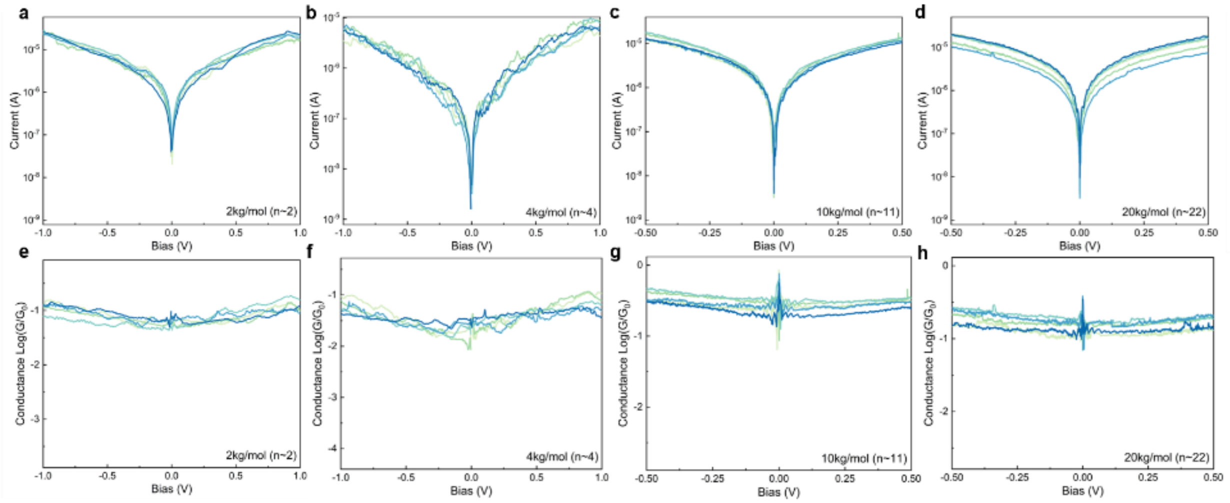

Figure S19. The representative I-V curves for open-shell macromolecules of a) 2 kg/mol, b) 4 kg/mol, c) 10 kg/mol, and d) 20 kg/mol collected once the junction is held at the position where the molecule is fully stretched. G-V curves calculated from representative I-V curves for e) 2 kg/mol, f) 4 kg/mol, g) 10 kg/mol, and h) 20 kg/mol.

4.3 Junction holding measurements

In this measurement, the tip retraction process was purposely controlled in repeated cycles, each with two phases. In the first phase, after the rupture of gold-gold atomic contact, the tip quickly withdrew by a distance equal to the most probable molecular plateau length determined by the corresponding 2D conductance *vs* displacement histogram measured earlier. This step allowed the trapped macromolecule to possess a fully extended configuration in the junction. In the second phase, the tip is held at a fixed position to stabilize the junction for a certain duration t (i.e., junction lifetime). After the holding time was reached, the tip continued to withdraw, typically resulting in a junction rupture immediately. Using this approach, we tested various junction holding times for 10 kg/mol ($n \sim 11$) and 20 kg/mol ($n \sim 22$) macromolecules, such as 100, 200, 300, and 500 seconds. Notably, the tested junctions remained intact for the tested holding time in this work, implying the junction lifetime could be longer than 500 seconds.

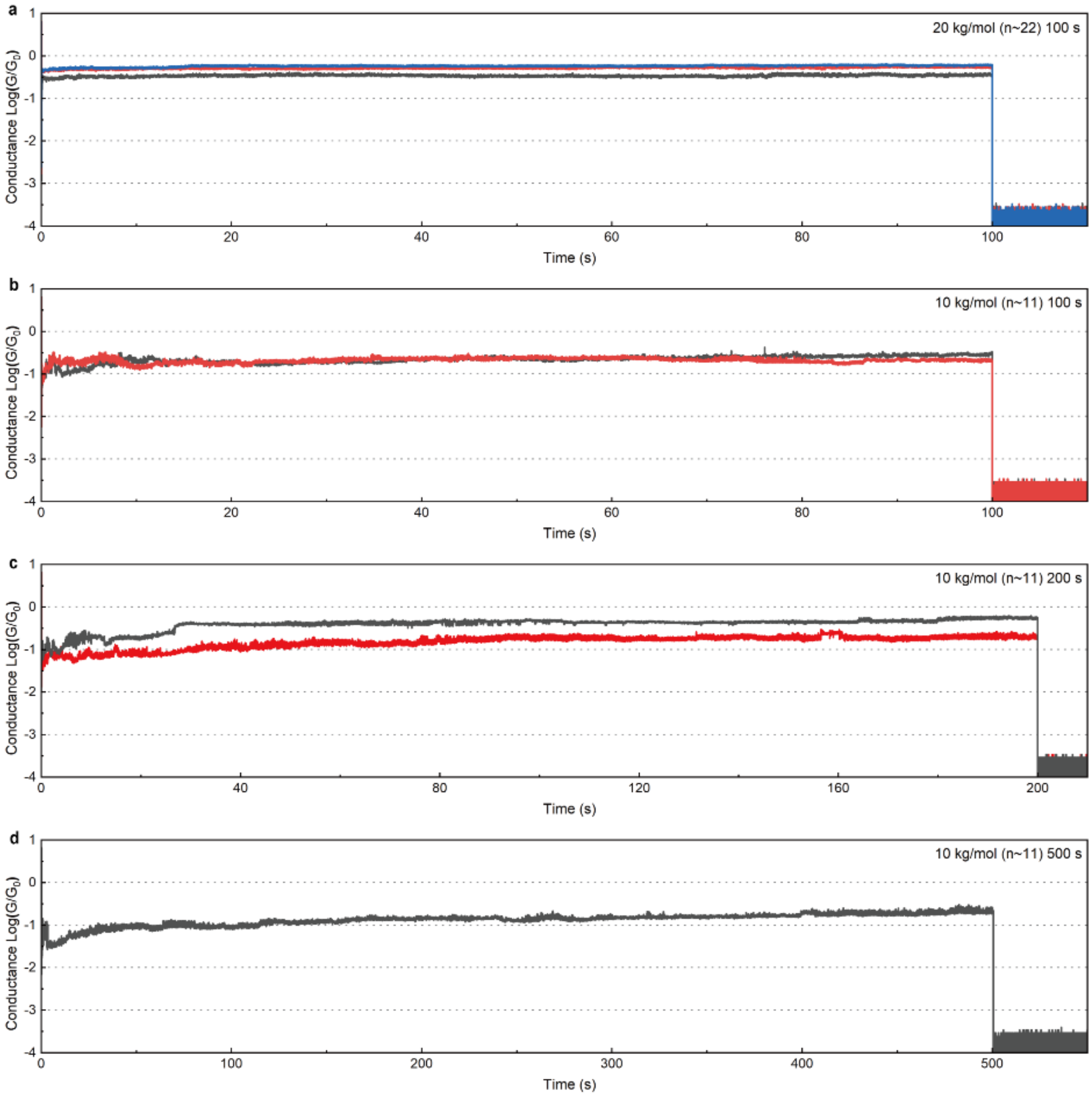

Figure S20. a) Example traces for open-shell 20 kg/mol (n ~ 22) macromolecular junctions mechanically stabilized for 100 seconds. b) Example traces for open-shell 10 kg/mol (n ~ 11) macromolecular junctions mechanically stabilized for b) 100, c) 200 and d) 500 seconds.

4.4 Mechanical modulation

We characterized the transport properties of a mechanically modulated macromolecular junction by varying the tip-substrate separation d by a distance of (Δd) in an STM-BJ setup, as detailed previously.[7] Specifically, after the rupture of gold-gold atomic contact, the tip was quickly

withdrawn by a distance (d) equal to the most probable molecular plateau length determined by the corresponding 2D conductance vs displacement histogram measured earlier. This step allowed the trapped macromolecule to possess a fully extended configuration in the junction prior to the subsequent mechanical modulation. Then, the tip position was modulated by a square waveform of amplitude Δd to repeatedly cycle through the fully extended state and a mechanically compressed state of the measured junction at a frequency of 2.5Hz for a duration of 1 second. We applied such mechanical modulation to 4 kg/mol (n ~ 4) and 20 kg/mol (n ~ 22) macromolecules. Here Δd of 2, 5, 8 Å were used for 4 kg/mol (n ~ 4) and Δd of 10, 20, 30, 40, 50 Å were used for 20 kg/mol (n ~ 22) macromolecule. High conductance (HG) and low conductance (LG) states were observed for the mechanically compressed state and fully extended state, respectively. After the mechanical modulation, further tip retraction was applied to rupture the junction and start the formation of a new molecular junction. The conductance (G) (current (I) / voltage (V)) was recorded throughout the tip retraction process under a static applied bias of 200mV. After collecting thousands of traces, we employed a custom-designed data processing algorithm to discard traces showing no evidence of junction formation after the initial tip retraction. The remaining traces were then compiled into 2D density maps and 1D histograms without data selection.

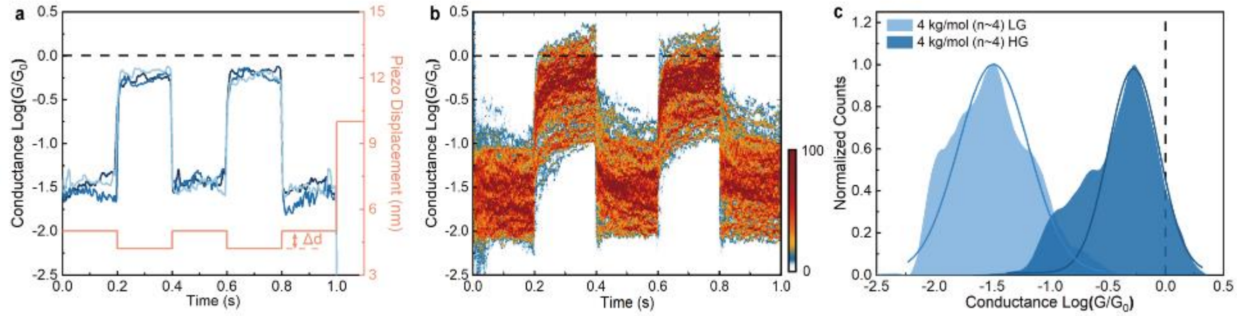

Figure S21. a) Representative conductance traces (blue) of mechanically modulated 4 kg/mol (n ~ 4) open-shell macromolecular junction with Δd = 8 Å. The red line represents the tip displacement curve. Under a junction compression, the conductance traces show reversible switching between high and low conductance states (HG and LG). The HG and LG states correspond to the compressed and fully extended junction configuration, respectively. The HG state is observed to rise close to $1G_0$. b) 2D conductance *vs.* time histograms of the 4 kg/mol (n ~ 4) open-shell macromolecule constructed from ~400 traces without data selection. c) 1D

conductance histograms of the HG (dark blue) and LG (light blue) plateaus for the data in b. Gaussian fitting to the data shows two distinctive conductance peaks for the fully extended LG state ($10^{-1.49}$ G_0) and compressed HG state ($10^{-0.28}$ G_0), revealing a 20-fold conductance enhancement upon an 8 Å mechanical compression of the junction.

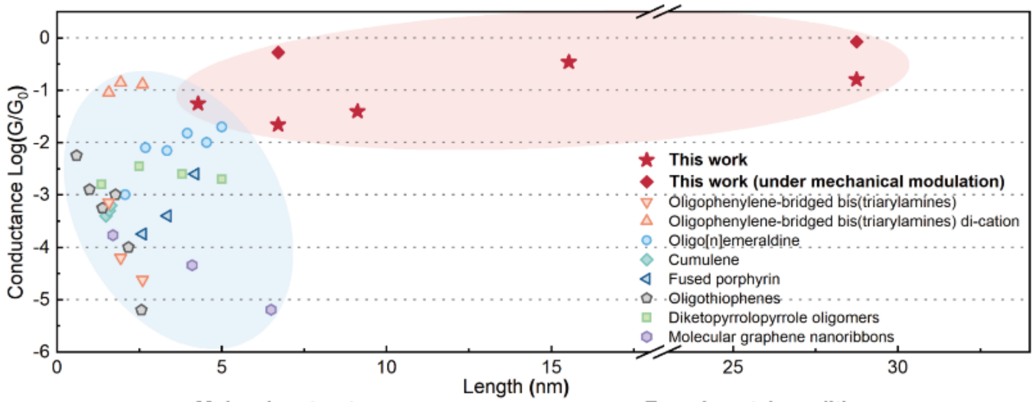

Molecular structure	Experimental conditions
★ ◆ This work 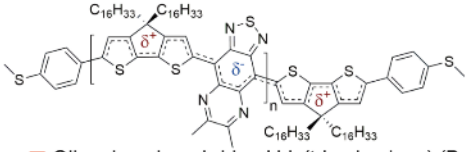	Bias voltage: 200 mV<br>Ambient condition in air
▽ Oligophenylene-bridegd bis(triarylamines) (Bn) 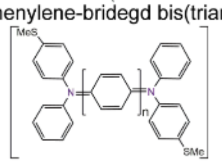	Bias voltage: 45 mV<br>Ambient condition in tert-butyl peroxyneo-caprate
△ Oligophenylene-bridegd bis(triarylamines) di-cation (Bn^{2+}) 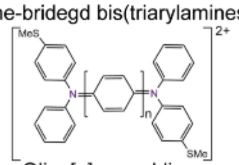	Bias voltage: 466 mV for $B1^{2+}$ and 379 mV for $B2^{2+}$ and $B3^{2+}$<br>Ambient condition in dichloromethane/tert-butyl peroxyneo-caprate
○ Oligo[n]emeraldine 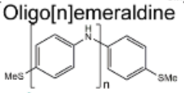	Bias voltage: 500 mV<br>Ambient condition in propylene carbonate/ trifluoroacetic acid
◇ Cumulene 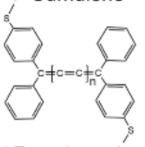	Bias voltage: 100 mV<br>Ambient condition in trichlorobenzene
◁ Fused porphyrin 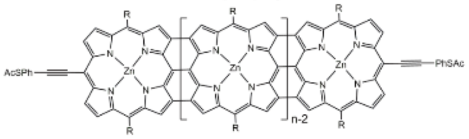	Bias voltage: 700 mV<br>Ambient condition in air
⬠ Oligothiophenes (Tn) 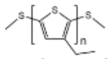	Bias voltage: 90 mV for T2, 220 mV for T3, T4 and T5, and 500 mV for T6<br>Ambient condition in trichlorobenzene
□ Diketopyrrolopyrrole oligomers 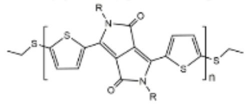	Bias voltage: 650 mV<br>Ambient condition in 1,2,4-trichlorobenzene
⬡ Molecular graphene nanoribbons (NR-n) 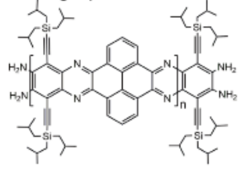	Bias voltage: 30 mV for NR-6, 200 mV for NR-16, and 300 mV for NR-26<br>Ambient condition in 1,3,5-trimethylbenzene

Figure S22. Comparison of the measured conductance of state-of-the-art π-conjugated molecules with the macromolecules reported in this work, as shown in **Figure 5** in the main text. For each work, the measured single-molecule conductance is plotted against the theoretically calculated molecular length reported in the study. The detailed experimental conditions for different molecular systems are provided for comparison as well. Solid data points represent experimental results obtained in air at ambient conditions in the low-bias regime (<300mV). Notably, not only do our macromolecules show the highest conductance and the longest transport length, but they are also the only molecular system capable of facilitating ultra-long-range resonant transport under low bias in air at ambient conditions. Molecules used in this comparison are oligophenylene-bridged bis(triarylamines)(neutral and dication),[8] oligo[n]emeraldine,[9] cumulene,[10] fused porphyrin,[11] oligothiophenes,[12] diketopyrrolopyrrole oligomers,[13] molecular graphene nanoribbons.[14]

4.5 Cyclic voltammetry

Electrochemical characteristics were determined by cyclic voltammetry (50 mV/s) on drop-cast polymer films at room temperature in degassed anhydrous acetonitrile with tetrabutylammonium hexafluorophosphate (0.1 M) as the supporting electrolyte. The working electrode was a platinum wire, the counter electrode was a platinum wire, and the reference electrode was Ag/AgCl in 3M KCl. The potential axis was calibrated to the reference electrode +0.21 V *vs.* the normal hydrogen electrode (NHE). Then, oxidation and reduction onsets were corrected by -4.44 eV for NHE *vs.* vacuum to determine ionization potentials and electron affinities. Δ demonstrates the difference between ionization potential and electron affinity energies.

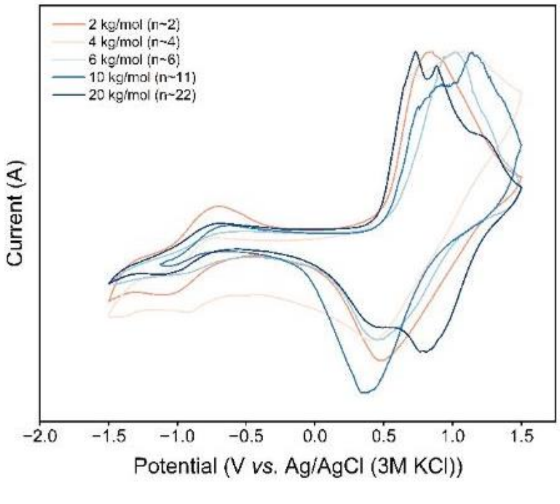

Figure S23. Cyclic voltammetry of a solid-film indicating the onset of oxidation and reduction as follows: (a) n = 2: -5.20 eV and -3.94 eV, respectively, Δ = 1.26 eV; (b) n = 4: -5.13 eV and -3.95 eV, respectively, Δ = 1.18 eV; (c) n = 6: -5.17 eV and -4.01 eV, respectively, Δ = 1.16 eV; (d) n = 11: -5.09 eV and -3.98 eV, respectively, Δ = 1.11 eV; (e) n = 22: -5.07 eV and -4.02 eV, respectively, Δ = 1.05 eV.

5. Theoretical Calculations

5.1 DFT Calculations.

DFT calculations on the model oligomer units were performed by progressively increasing the size of the π-system from n = 1 to 8 using the Gaussian 16 software package. Hexadecyl ($–C_{16}H_{33}$) side chains of the donor were truncated with methyl ($–CH_3$) groups. All energy-minimized geometries obtained from semi-empirical methods (PM6) were subjected to optimization calculations using the spin-restricted Becke's three-parameter (B3LYP) density functional and 6-31G** basis set.[15] Broken-symmetry (BS) formalism was adopted with a restricted wave function and applied to generate the initial guess wave function with a 1:1 mixture to remove α-β and spatial symmetries, with ⟨S2⟩ values that are different from 0 (pure singlet) and 2 (pure triplet). However, wave functions corresponding to oligomers with a π-conjugation length of n = 4 to 8 showed restricted-to-unrestricted instability, and were further subjected to wave function stability test, providing a stable wave function with singlet-triplet electronic ground states at the unrestricted B3LYP/6-31G** level of theory. The diradical character index (y) at the same level of theory has been evaluated from the highest occupied natural orbital (HONO) and lowest unoccupied natural orbital (LUNO) occupancies by following Yamaguchi's formula (Eq. S3)

$$y = 1 - \frac{T}{1+T^2} \qquad \text{(Eq. S3)}$$

where T is defined as the orbital overlap that can be calculated using the occupation numbers (n) obtained from the unrestricted natural orbitals in equation (Eq. S4):

$$T = \frac{n_{HONO} - n_{LUNO}}{2} \quad \text{(Eq. S4)}$$

Spin locations were predicted from the natural spin densities of Kohn-Sham molecular orbitals (MO). Molecular electrostatic potential surface (ESP) and FMOs involved in electron and spin density distribution analyses were calculated using the UB3LYP/6-311G** level of theory. NICS$_{iso}$(1) calculations were performed using the gauge-independent atomic orbital (GIAO) method on the BS optimized geometry to assess the effect of the ring current produced by the π-electrons from each ring of the model oligomers. To diminish contributions of π-bonding contributions to the π-ring current, a so-called ghost atom (B_q) was placed 1 Å perpendicular to the ring plane. The obtained values have been generally reported as the negative value of the absolute isotropic magnetic shielding, where large negative NICS values indicates a more pronounced aromaticity containing (4n+2) π electrons, while the smaller negative values suggest an involvement of quinoidal characteristics with 4n π-electrons.

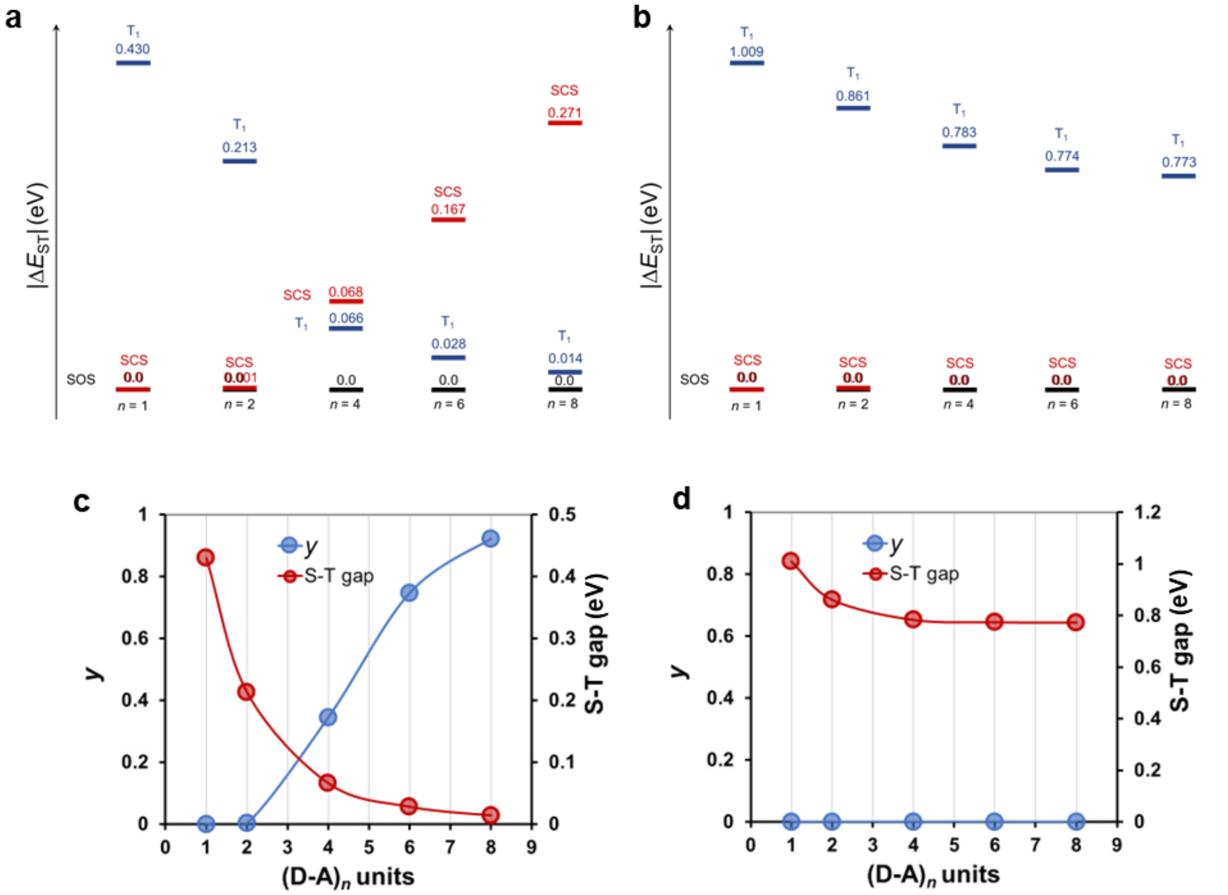

Figure S24. a) Absolute value of ΔE_{ST} of open-shell macromolecules as a function of n = 1, 2, 4, 6, and 8 repeating units showing the change in relative energies of the various spin states with reference to singlet open-shell (SOS) state. T_1: Triplet state; SCS: singlet closed shell. b) Absolute value of ΔE_{ST} of closed-shell macromolecules as a function of n = 1, 2, 4, 6, and 8 repeating units showing the change in relative energies of the various spin states with reference to singlet open-shell (SOS) state. T_1: Triplet state; SCS: singlet closed shell. Diradical character index (y_0) of c) open-shell and d) closed-shell macromolecules plotted against the absolute value of ΔE_{ST} for the oligomers with n = 1-8 repeating units.

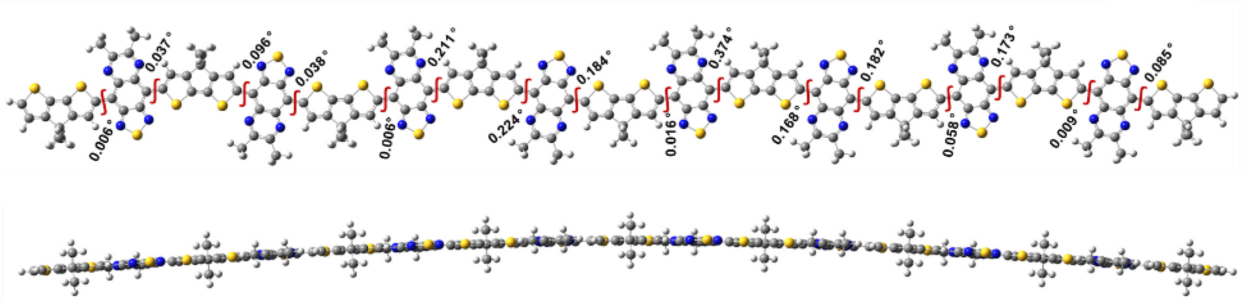

Figure S25. The optimized geometries of the octamer (n = 8) corresponding to the open-shell macromolecule with selected dihedral angles and enhanced planarity of the π-framework that can be seen from the side perspective.

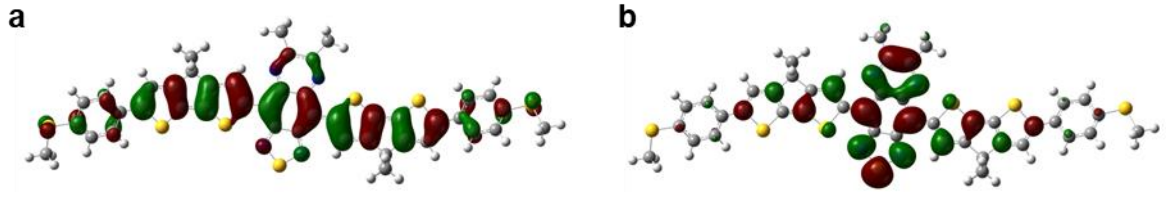

Figure S26. Optimized closed-shell ground state geometric structures for the single (n = 1) repeated unit of open-shell macromolecule and pictorial representations of the frontier molecular orbitals. a) HOMO, and b) LUMO. The green and red surfaces represent positive and negative signs of the MO at isovalue = 0.02 au, respectively. Color codes for the atoms are gray for C, blue for N and yellow for S.

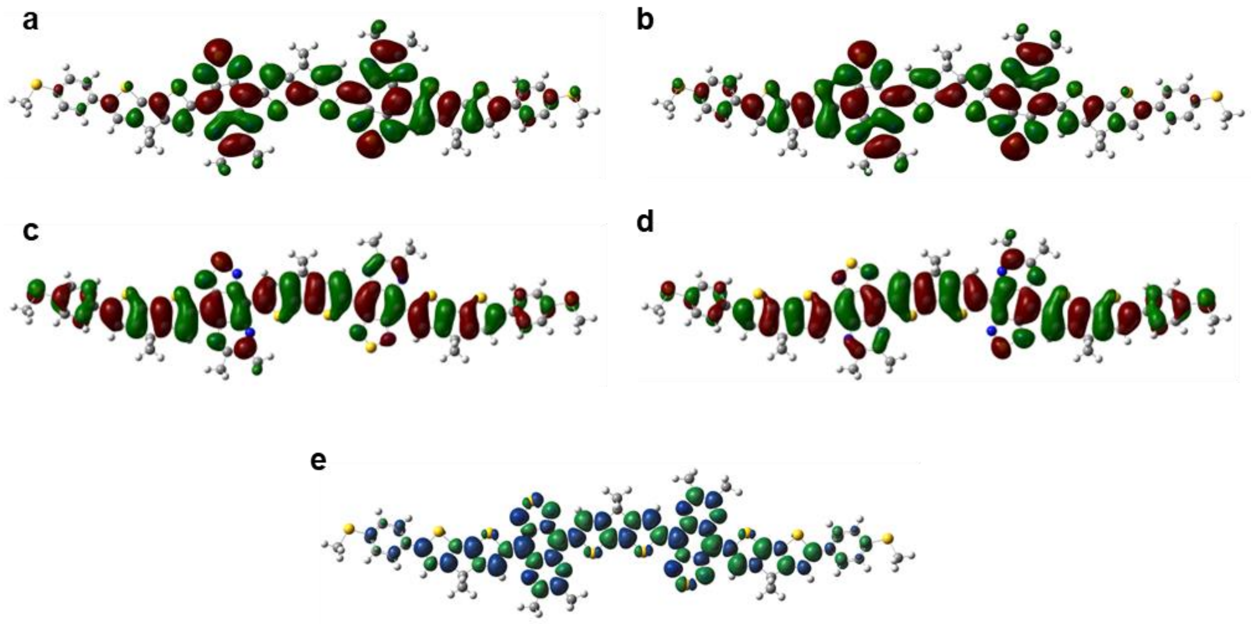

Figure S27. Optimized ground state geometric structures for the dimer (n = 2) repeating units of open-shell macromolecule and pictorial representations of the frontier molecular orbitals and spin density distribution. a) α-SUMO and b) β-SUMO, c) α-SOMO and d) β-SOMO, and e) spin density distribution of the open-shell singlet. The green and red surfaces represent positive and negative signs of the molecular orbital at isovalue = 0.02 au, respectively. The blue and green surfaces represent positive and negative contributions of the spin density at an isovalue = 0.0004 au. Color codes for the atoms are gray for C, blue for N and yellow for S.

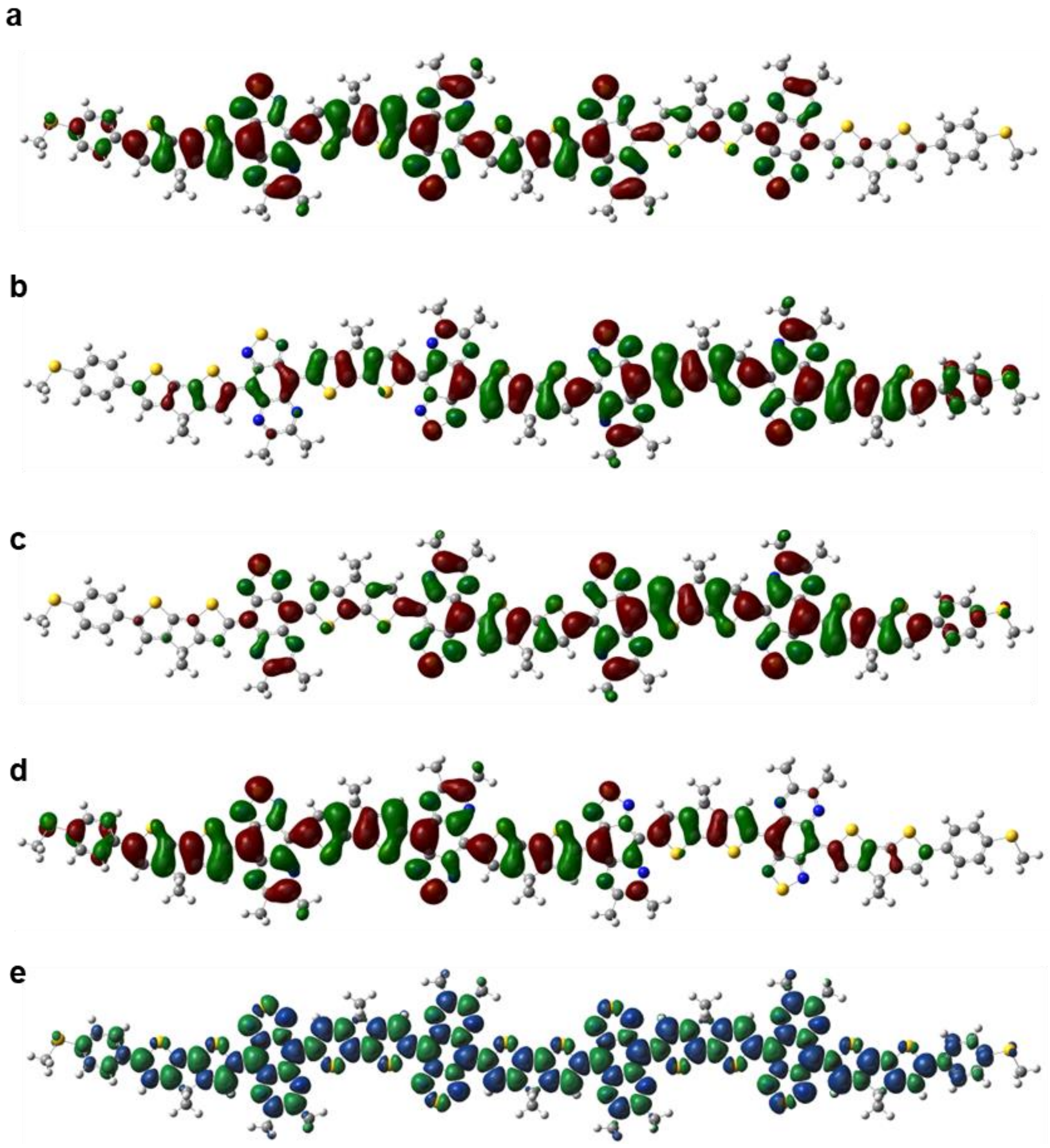

Figure S28. Optimized ground state geometric structures for the tetramer (*n* = 4) repeating units of open-shell macromolecule and pictorial representations of the frontier molecular orbitals and spin density distribution. a) α-SUMO and b) β-SUMO, c, α-SOMO and d) β-SOMO, and e) the spin density distribution of the open-shell singlet. The green and red surfaces represent positive and negative signs of the molecular orbital at isovalue = 0.02 au, respectively. The blue and green surfaces represent positive and negative contributions of the spin density at an isovalue = 0.0004 au. Color codes for the atoms are gray for C, blue for N and yellow for S.

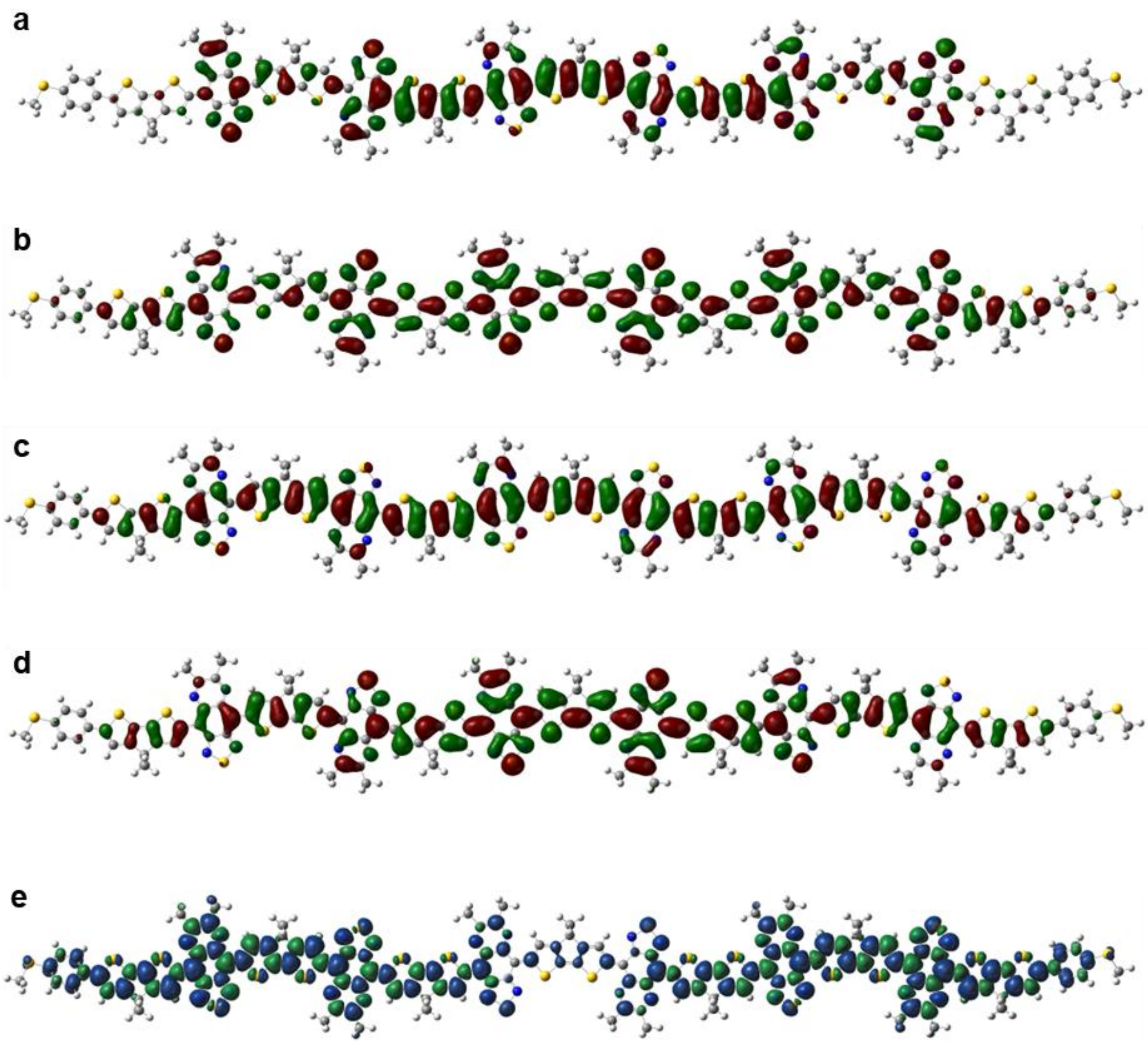

Figure S29. Optimized ground state geometric structures for the hexamer (n = 6) repeating units of open-shell macromolecule and pictorial representations of the frontier molecular orbitals and spin density distribution. a) α-SUMO and b) β-SUMO, c) α-SOMO and d) β-SOMO, and e) the spin density distribution of the open-shell triplet. The green and red surfaces represent positive and negative signs of the molecular orbital at isovalue = 0.02 au, respectively. The blue and green surfaces represent positive and negative contributions of the spin density at an isovalue = 0.0004 au. Color codes for the atoms are gray for C, blue for N and yellow for S.

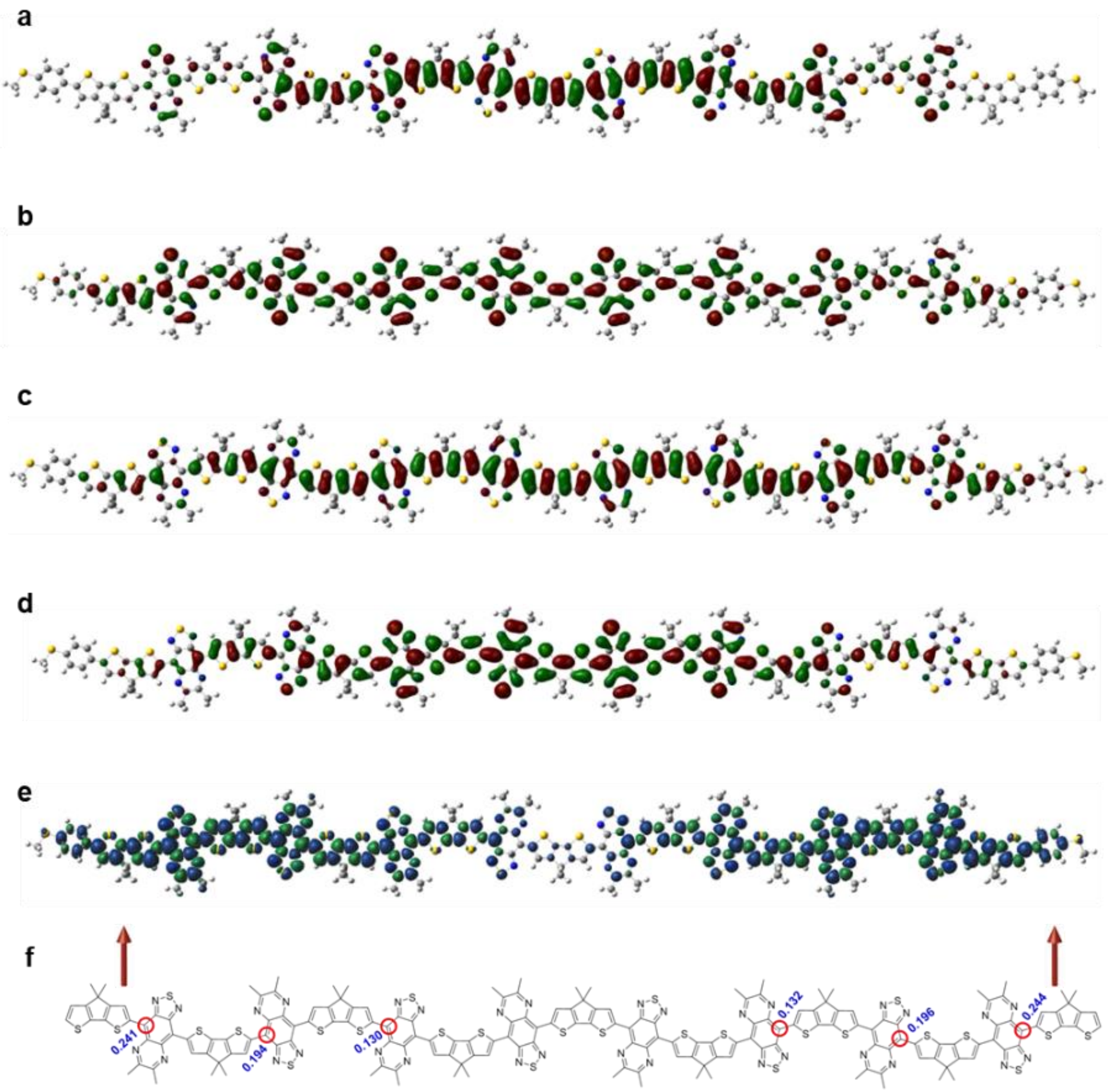

Figure S30. Optimized ground state geometric structures for the octamer (*n* = 8) repeating units of open-shell macromolecule and pictorial representations of the frontier molecular orbitals and spin density distribution. a) α-SUMO and b) β-SUMO, c) α-SOMO and d) β-SOMO, and e) the spin density distribution of the open-shell triplet. The green and red surfaces represent positive and negative signs of the molecular orbital at isovalue = 0.02 au, respectively. The blue and green surfaces represent positive and negative contributions of the spin density at an isovalue = 0.0004 au. Color codes for the atoms are gray for C, blue for N and yellow for S. f) Red circles mark the spin centers with maximum spin density coefficients, highlighting the localization of unpaired electrons at the terminal repeating units in the *n* = 8 of open-shell macromolecule triplet ground state geometry.

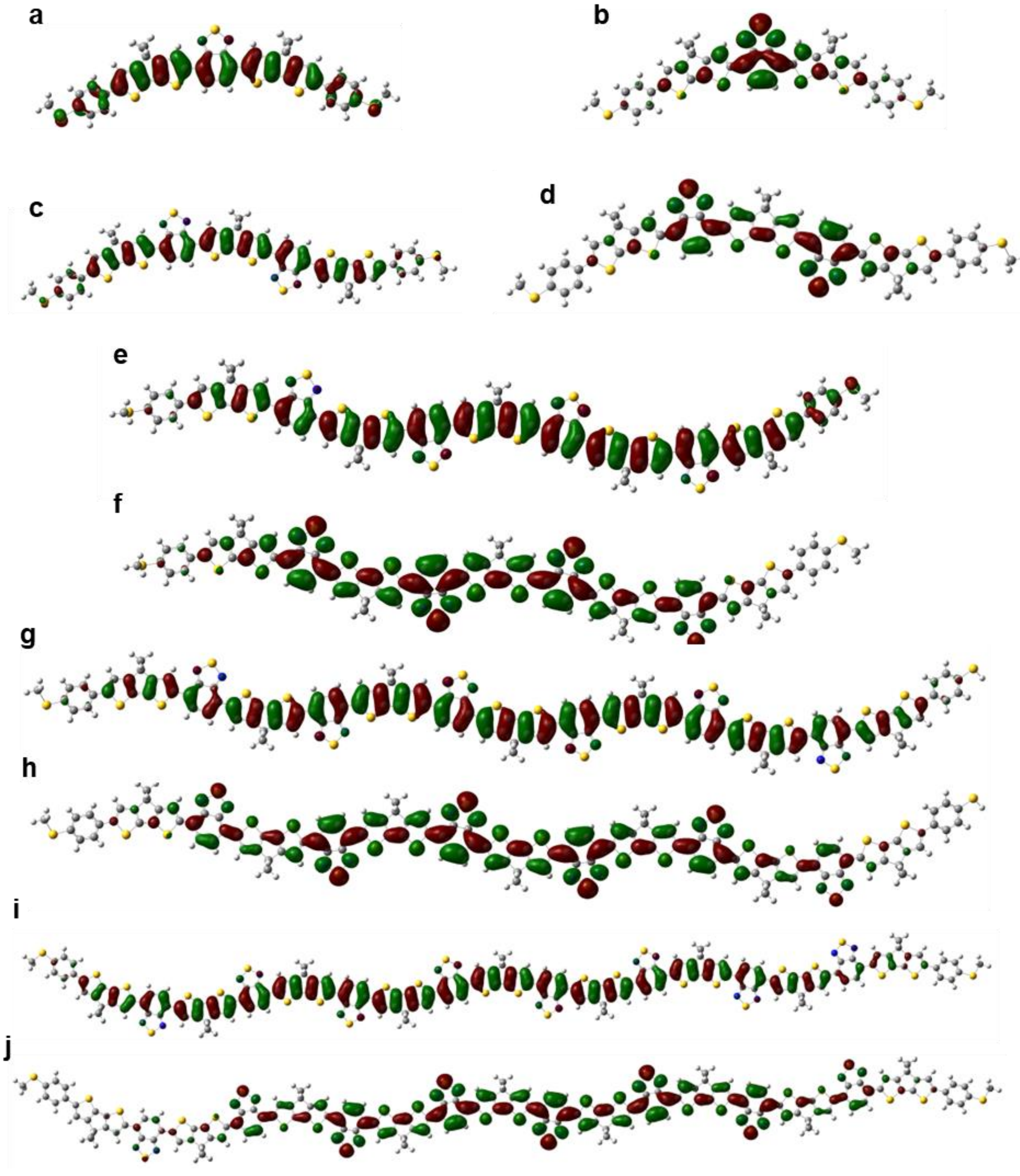

Figure S31. Optimized closed-shell ground state geometric structures for a) (*n* = 1) HOMO b) (*n* = 1) LUMO, c) (*n* = 2) HOMO, d) (*n* = 2) LUMO, e) (*n* = 4) HOMO, f) (*n* = 4) LUMO, g) (*n* = 6) HOMO, h) (*n* = 6) LUMO, i) (*n* = 8) HOMO, j) (*n* = 8) LUMO of closed-shell control macromolecule and pictorial representations of the frontier molecular orbitals. The green and red

surfaces represent positive and negative signs of the molecular orbital at isovalue = 0.02 au, respectively. Color codes for the atoms are gray for C, blue for N and yellow for S.

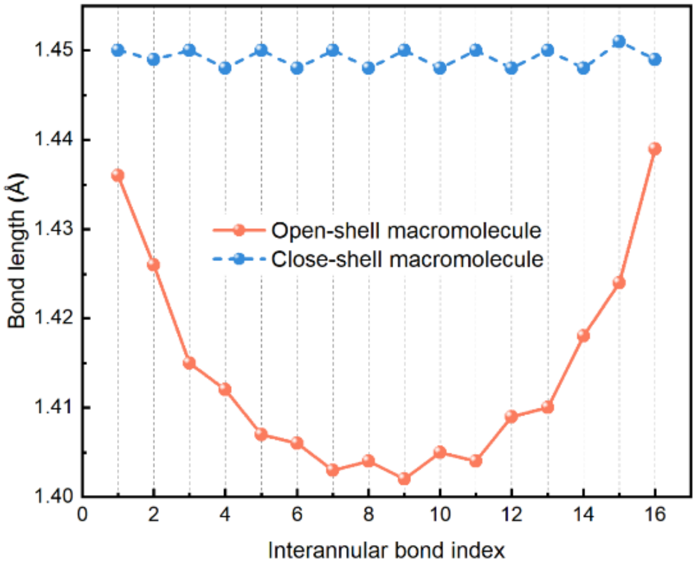

Figure S32. Interannular bond distance between the donor and acceptor segments from the optimized octamer geometries corresponding to open-shell and closed-shell macromolecules.

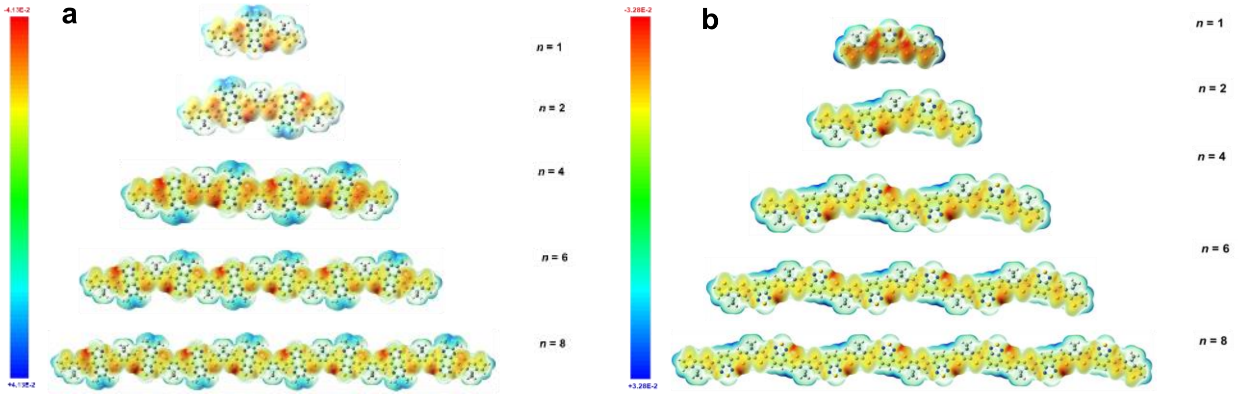

Figure S33. a) Molecular electrostatic potential (MESP) surface plots of the optimized oligomers with n = 1, 2, 4, 6, and 8 repeating units corresponding to open-shell macromolecule reinforcing the existence of intramolecular H-bonding and S···N interactions through a weak to the moderate negative electrostatic environment between the DA units. b) MESP surface plots of the optimized oligomers with n = 1, 2, 4, 6, and 8 repeating units corresponding to closed-shell control macromolecule displaying the existence of S···N interactions through a moderate negative electrostatic environment between the DA units.

Table S3. Selected electronic properties as a function of the number of repeating units *n* for the open-shell and closed-shell macromolecules.

n[a] (Open-shell)	ΔE_{ST}[b] (eV)	*n*HONO[c]	*n*LUNO[c]	y_0	SOS-SCS (eV)	μ_g[d] (Debye)
**1**	0.430	2	0	0	0	2.6
**2**	0.213	1.9259	0.0741	0.003	0.001	1.91
**4**	0.066	1.3733	0.6268	0.345	0.068	2.2
**6**	0.028	1.1287	0.8714	0.747	0.167	2.96
**8**	0.014	1.0389	0.9611	0.922	0.271	3.78

n[a] (Closed-shell)	ΔE_{ST}[b] (eV)	*n*HONO[c]	*n*LUNO[c]	y_0	SOS-SCS (eV)	μ_g[d] (Debye)
**1**	0.430	2	0	0	0	2.05
**2**	0.213	2	0	0	0	1.22
**4**	0.066	2	0	0	0	1.91
**6**	0.028	2	0	0	0	3.15
**8**	0.014	2	0	0	0	4.55

[a]Number of repeating units (*n*) for the calculated oligomers. [b]Singlet^{BS}-triplet energy gap. [c]Natural orbital occupancies, [d]diradical character index (y_0) calculated from Yamaguchi's formula, and [d]ground state dipole moment as determined at the (U)B3LYP/6-31G** level of theory. Natural orbital occupancies (HONO, LUNO) and y_0 are unitless quantities. SCS: singlet closed shell; SOS: singlet open shell.

Table S4. Tabulated NICS values of the single repeating unit (*n* = 1) for open-shell and closed-shell macromolecules.

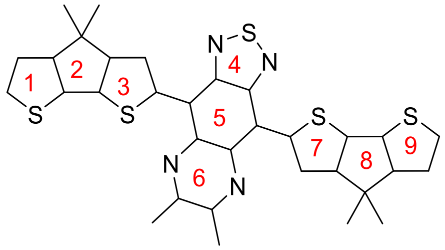

Ring Index	Open-shell [*n*=1]	Closed-shell [*n*=1]
**1**	-7.68	-11.23
**2**	-2.75	-4.55
**3**	-6.45	-9.62
**4**	-11.78	-12.8

**5**	-10.65	-11.44
**6**	-9.33	-
**7**	-7.32	-7.56
**8**	-3.45	-4.32
**9**	-6.13	-7.11

Table S5. Tabulated NICS values of the two repeating units ($n = 2$) for open-shell and closed-shell macromolecules.

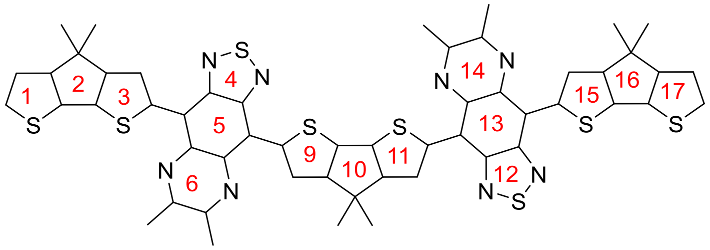

Ring Index	Open-shell [*n*=2]	Closed-shell [*n*=2]	Ring Index	Open-shell [*n*=2]	Closed-shell [*n*=2]
**1**	-7.44	-10.19	**10**	-5.56	-7.55
**2**	-0.11	-1.89	**11**	-0.58	-12.71
**3**	-5.68	-7.39	**12**	-13.8	-7.00
**4**	13.31	-13.46	**13**	-9.13	-1.83
**5**	-7.21	-6.50	**14**	-5.95	-
**6**	-8.23	-	**15**	-7.82	-7.80
**7**	-7.44	-7.59	**16**	-5.56	-13.58
**8**	0.11	-1.56	**17**	-0.58	-6.28
**9**	-5.68	-10.08			

Table S6. Tabulated NICS values of the four repeating units ($n = 4$) for open-shell and closed-shell macromolecules.

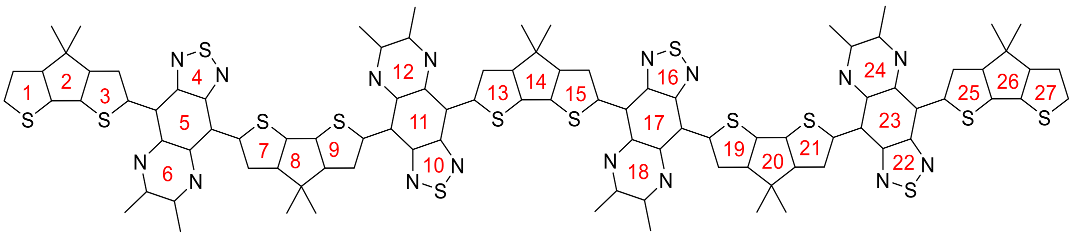

Ring Index	Open-shell [*n*=4]	Closed-shell [*n*=4]	Ring Index	Open-shell [*n*=4]	Closed-shell [*n*=4]	Ring Index	Open-shell [*n*=4]	Closed-shell [*n*=4]
**1**	-7.59	-10.65	**10**	-10.94	-6.85	**19**	-5.19	-7.25
**2**	-0.46	-2.35	**11**	-3.19	-13.78	**20**	-1.14	-2.05
**3**	-5.73	-7.85	**12**	-8.76	-	**21**	-5.85	-7.97
**4**	-11.98	-13.92	**13**	-5.99	-7.65	**22**	-13.6	-13.29
**5**	-5.28	-6.96	**14**	-1.91	-1.96	**23**	-8.71	-6.81
**6**	-8.79	10.65	**15**	-5.62	-8.26	**24**	-7.98	-
**7**	-5.21	-7.54	**16**	-11.52	-13.95	**25**	-5.84	-7.82
**8**	-1.63	-2.4	**17**	-4.18	-6.54	**26**	-1.76	-1.79
**9**	-5.29	-8.42	**18**	-8.47	-	**27**	-5.47	-10.31

Table S7. Tabulated NICS values of the six repeating units (n = 6) for open-shell and closed-shell macromolecules.

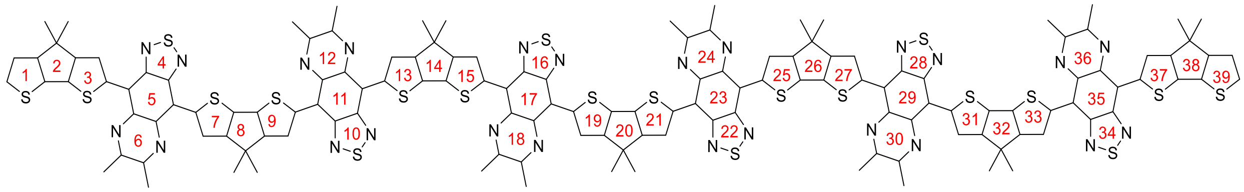

Ring Index	Open-shell [*n*=6]	Closed-shell [*n*=6]	Ring Index	Open-shell [*n*=6]	Closed-shell [*n*=6]	Ring Index	Open-shell [*n*=6]	Closed-shell [*n*=6]
**1**	-7.44	-10.42	**14**	-1.46	-2.06	**27**	-4.66	-8.26
**2**	-0.31	-2.12	**15**	-4.17	-8.03	**28**	-11.52	-13.95
**3**	-5.58	-7.62	**16**	-10.34	-13.81	**29**	-4.18	-6.54
**4**	-11.83	-13.69	**17**	-2.24	-6.51	**30**	-8.47	-
**5**	-5.13	-6.73	**18**	-8.69	-	**31**	-5.19	-7.25
**6**	-8.64	-	**19**	-4.34	-7.28	**32**	-1.14	-2.05
**7**	-5.06	-7.31	**20**	-1.9	-2.06	**33**	-5.85	-7.97
**8**	-1.48	-2.17	**21**	-4.88	-7.78	**34**	-13.6	-13.29
**9**	-5.14	-8.19	**22**	-10.83	-12.94	**35**	-8.71	-6.81
**10**	-10.79	-6.62	**23**	-2.94	-6.68	**36**	-7.98	-
**11**	-3.04	-13.55	**24**	-8.61	-	**37**	-5.84	-7.82

**12**	-8.61	-	**25**	-4.56	-7.65	**38**	-1.76	-1.79
**13**	-4.16	-7.23	**26**	-1.14	-1.96	**39**	-5.47	-10.31

Table S8. Tabulated NICS values of the eight repeating units (*n* = 8) for open-shell and closed-shell macromolecules.

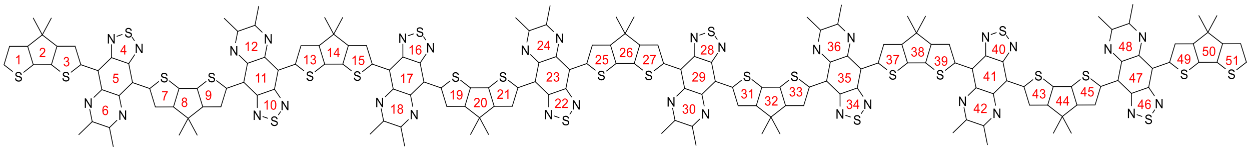

**Ring Index**	**Open-shell [*n*=8]**	**Closed-shell [*n*=8]**	**Ring Index**	**Open-shell [*n*=8]**	**Closed-shell [*n*=8]**	**Ring Index**	**Open-shell [*n*=8]**	**Closed-shell [*n*=8]**	**Ring Index**	**Open-shell [*n*=8]**	**Closed-shell [*n*=8]**
**1**	-7.29	-10.19	**17**	-2.14	-6.41	**33**	-4.73	-7.55	**49**	-5.69	-7.59
**2**	-0.16	-1.89	**18**	-8.49	-	**34**	-10.68	-12.71	**50**	-1.61	-1.56
**3**	-5.43	-7.39	**19**	-4.04	-6.92	**35**	-2.79	-6.45	**51**	-5.32	-10.08
**4**	-11.68	-13.46	**20**	-1.75	-1.83	**36**	-8.46	-			
**5**	-4.98	-6.50	**21**	-4.33	-7.85	**37**	-4.41	-7.42			
**6**	-8.49	-	**22**	-10.07	-13.98	**38**	-0.99	-1.73			
**7**	-4.91	-7.08	**23**	-1.78	-6.43	**39**	-4.51	-8.03			
**8**	-1.33	-1.94	**24**	-8.51	-	**40**	-11.37	-13.72			
**9**	-4.99	-7.96	**25**	-4.01	-7.00	**41**	-4.03	-6.31			
**10**	-10.64	-6.39	**26**	-1.31	-1.83	**42**	-8.32	-			
**11**	-2.89	-13.32	**27**	-4.02	-7.80	**43**	-5.04	-7.02			
**12**	-8.46	-	**28**	-10.19	-13.58	**44**	-0.99	-1.82			
**13**	-4.31	-7.09	**29**	-2.09	-6.28	**45**	-5.70	-7.74			
**14**	-1.19	-1.91	**30**	-8.54	-	**46**	-13.45	-13.06			
**15**	-4.14	-7.70	**31**	-4.19	-7.05	**47**	-8.56	-6.58			
**16**	-10.20	-13.50	**32**	-1.75	-1.83	**48**	-7.83	-			

Table S9. Tabulated bond length values (Å) of the single repeating unit (*n* = 1) for open-shell and closed-shell macromolecules.

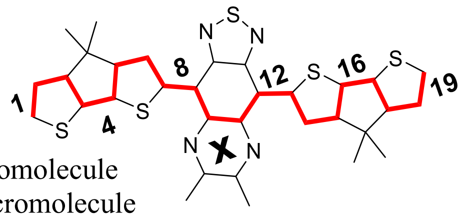

X = 1, Open-shell macromolecule
X = 0, Closed-shell macromolecule

Bond Index	Open-shell [*n*=1]	Closed-shell [*n*=1]	Bond Index	Open-shell [*n*=1]	Closed-shell [*n*=1]
**1**	1.374	1.373	**11**	1.451	1.390
**2**	1.418	1.419	**12**	1.422	1.451
**3**	1.387	1.386	**13**	1.447	1.390
**4**	1.437	1.439	**14**	1.401	1.409
**5**	1.389	1.386	**15**	1.401	1.386
**6**	1.400	1.409	**16**	1.389	1.439
**7**	1.401	1.390	**17**	1.437	1.386
**8**	1.450	1.451	**18**	1.387	1.419
**9**	1.435	1.390	**19**	1.418	1.373
**10**	1.427	1.409			

Table S10. Tabulated bond length values (Å) of the two repeating units ($n = 2$) for open-shell and closed-shell macromolecules.

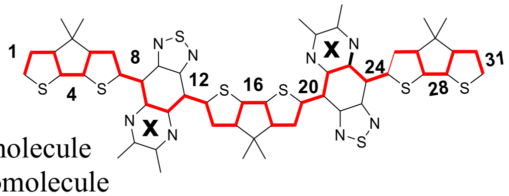

X = 1, Open-shell macromolecule
X = 0, Closed-shell macromolecule

Bond Index	Open-shell [*n*=2]	Closed-shell [*n*=2]	Bond Index	Open-shell [*n*=2]	Closed-shell [*n*=2]
**1**	1.374	1.374	**17**	1.399	1.392
**2**	1.418	1.419	**18**	1.392	1.402
**3**	1.388	1.386	**19**	1.408	1.396
**4**	1.436	1.438	**20**	1.442	1.451
**5**	1.390	1.386	**21**	1.433	1.393
**6**	1.399	1.409	**22**	1.449	1.408
**7**	1.403	1.390	**23**	1.425	1.392
**8**	1.447	1.450	**24**	1.444	1.450
**9**	1.431	1.391	**25**	1.403	1.390

**10**	1.449	1.408	**26**	1.400	1.409
**11**	1.427	1.391	**27**	1.390	1.386
**12**	1.440	1.449	**28**	1.436	1.438
**13**	1.408	1.392	**29**	1.388	1.386
**14**	1.393	1.407	**30**	1.418	1.419
**15**	1.398	1.39	**31**	1.374	1.373
**16**	1.422	1.432			

Table S11. Tabulated bond length values (Å) of the four repeating units (n = 4) for open-shell and closed-shell macromolecules.

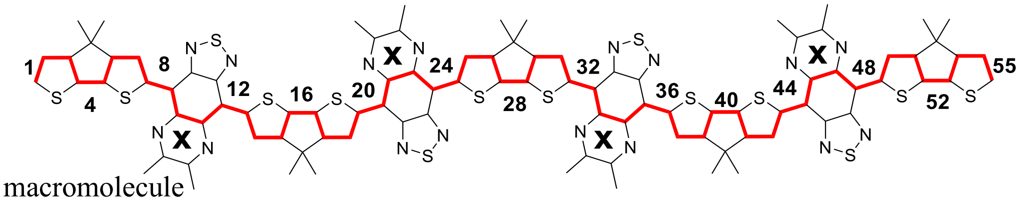

X = 1, Open-shell macromolecule
X = 0, Closed-shell macromolecule

Bond Index	Open-shell [*n*=4]	Closed-shell [*n*=4]	Bond Index	Open-shell [*n*=4]	Closed-shell [*n*=4]	Bond Index	Open-shell [*n*=4]	Closed-shell [*n*=4]
**1**	1.375	1.374	**20**	1.421	1.450	**39**	1.411	1.390
**2**	1.417	1.419	**21**	1.443	1.393	**40**	1.408	1.432
**3**	1.389	1.386	**22**	1.441	1.407	**41**	1.409	1.392
**4**	1.434	1.438	**23**	1.451	1.393	**42**	1.384	1.402
**5**	1.392	1.386	**24**	1.421	1.448	**43**	1.418	1.396
**6**	1.397	1.409	**25**	1.425	1.392	**44**	1.427	1.451
**7**	1.406	1.390	**26**	1.378	1.406	**45**	1.436	1.393
**8**	1.438	1.450	**27**	1.414	1.390	**46**	1.445	1.408
**9**	1.433	1.391	**28**	1.402	1.432	**47**	1.439	1.392
**10**	1.445	1.408	**29**	1.414	1.392	**48**	1.441	1.449
**11**	1.443	1.391	**30**	1.379	1.402	**49**	1.407	1.390
**12**	1.430	1.449	**31**	1.424	1.396	**50**	1.396	1.409
**13**	1.418	1.392	**32**	1.418	1.450	**51**	1.393	1.386
**14**	1.383	1.407	**33**	1.444	1.393	**52**	1.434	1.438
**15**	1.409	1.390	**34**	1.441	1.407	**53**	1.389	1.386
**16**	1.407	1.432	**35**	1.450	1.394	**54**	1.417	1.419
**17**	1.410	1.392	**36**	1.423	1.448	**55**	1.375	1.374

**18**	1.382	1.402	**37**	1.422	1.392
**19**	1.421	1.396	**38**	1.381	1.406

Table S12. Tabulated bond length values (Å) of the six repeating units (*n* = 6) for open-shell and closed-shell macromolecules.

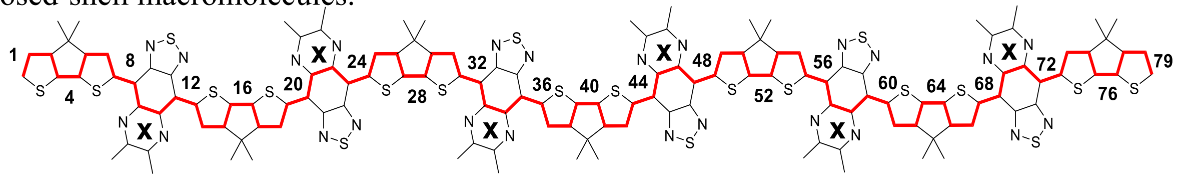

X = 1, Open-shell macromolecule
X = 0, Closed-shell macromolecule

Bond Index	Open-shell [*n*=6]	Closed-shell [*n*=6]	Bond Index	Open-shell [*n*=6]	Closed-shell [*n*=6]	Bond Index	Open-shell [*n*=6]	Closed-shell [*n*=6]
**1**	1.375	1.374	**29**	1.424	1.406	**57**	1.428	1.407
**2**	1.417	1.419	**30**	1.369	1.392	**58**	1.376	1.393
**3**	1.389	1.386	**31**	1.435	1.448	**59**	1.416	1.450
**4**	1.433	1.438	**32**	1.404	1.394	**60**	1.402	1.396
**5**	1.393	1.386	**33**	1.438	1.407	**61**	1.413	1.402
**6**	1.396	1.409	**34**	1.367	1.393	**62**	1.380	1.392
**7**	1.408	1.390	**35**	1.428	1.450	**63**	1.391	1.432
**8**	1.436	1.449	**36**	1.385	1.396	**64**	1.423	1.390
**9**	1.435	1.444	**37**	1.427	1.402	**65**	1.393	1.407
**10**	1.444	1.392	**38**	1.367	1.392	**66**	1.422	1.392
**11**	1.447	1.408	**39**	1.437	1.432	**67**	1.422	1.449
**12**	1.424	1.393	**40**	1.402	1.390	**68**	1.439	1.391
**13**	1.423	1.451	**41**	1.455	1.406	**69**	1.444	1.408
**14**	1.379	1.396	**42**	1.435	1.392	**70**	1.442	1.391
**15**	1.414	1.402	**43**	1.463	1.448	**71**	1.438	1.450
**16**	1.401	1.392	**44**	1.406	1.394	**72**	1.408	1.390
**17**	1.415	1.432	**45**	1.436	1.407	**73**	1.395	1.409
**18**	1.377	1.390	**46**	1.369	1.393	**74**	1.393	1.386
**19**	1.427	1.406	**47**	1.425	1.450	**75**	1.433	1.438
**20**	1.44	1.392	**48**	1.389	1.396	**76**	1.389	1.386
**21**	1.449	1.448	**49**	1.424	1.402	**78**	1.417	1.419
**22**	1.438	1.393	**50**	1.371	1.392	**79**	1.375	1.374
**23**	1.459	1.450	**51**	1.433	1.432			

**24**	1.409	1.396	**52**	1.408	1.390
**25**	1.434	1.402	**53**	1.451	1.406
**26**	1.370	1.392	**54**	1.438	1.392
**27**	1.424	1.432	**55**	1.457	1.448
**28**	1.389	1.390	**56**	1.416	1.393

Table S13. Tabulated bond length values (Å) of the eight repeating units (n = 8) for open-shell and closed-shell macromolecules.

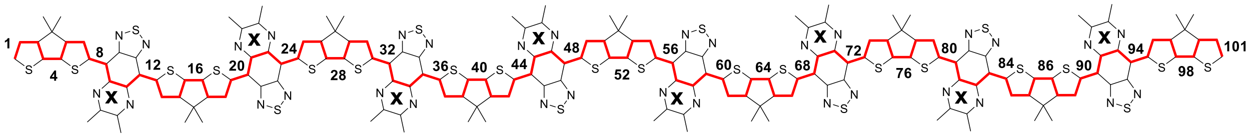

X = 1, Open-shell macromolecule
X = 0, Closed-shell macromolecule

**Bond Index**	**Open-shell [*n*=8]**	**Closed-shell [*n*=8]**	**Bond Index**	**Open-shell [*n*=8]**	**Closed-shell [*n*=8]**	**Bond Index**	**Open-shell [*n*=8]**	**Closed-shell [*n*=8]**
**1**	1.375	1.374	**37**	1.426	1.396	**73**	1.434	1.396
**2**	1.417	1.419	**38**	1.387	1.402	**74**	1.371	1.402
**3**	1.389	1.386	**39**	1.426	1.396	**75**	1.423	1.392
**4**	1.396	1.438	**40**	1.368	1.431	**76**	1.392	1.432
**5**	1.407	1.386	**41**	1.437	1.390	**77**	1.421	1.390
**6**	1.436	1.409	**42**	1.403	1.406	**78**	1.373	1.406
**7**	1.435	1.390	**43**	1.455	1.392	**79**	1.431	1.392
**8**	1.444	1.449	**44**	1.435	1.448	**80**	1.410	1.448
**9**	1.446	1.392	**45**	1.464	1.394	**81**	1.449	1.393
**10**	1.426	1.408	**46**	1.404	1.407	**82**	1.438	1.450
**11**	1.421	1.393	**47**	1.438	1.393	**83**	1.455	1.396
**12**	1.380	1.451	**48**	1.367	1.450	**84**	1.418	1.402
**13**	1.413	1.396	**49**	1.428	1.396	**85**	1.426	1.392
**14**	1.403	1.402	**50**	1.385	1.402	**86**	1.378	1.432
**15**	1.414	1.392	**51**	1.427	1.392	**87**	1.414	1.390
**16**	1.378	1.432	**52**	1.367	1.432	**88**	1.403	1.407
**17**	1.426	1.390	**53**	1.438	1.390	**89**	1.412	1.392
**18**	1.415	1.406	**54**	1.402	1.406	**90**	1.381	1.449
**19**	1.447	1.392	**55**	1.455	1.392	**91**	1.421	1.391

**20**	1.438	1.448	**56**	1.435	1.448	**92**	1.424	1.408
**21**	1.457	1.394	**57**	1.464	1.394	**93**	1.438	1.391
**22**	1.412	1.407	**58**	1.405	1.407	**94**	1.444	1.450
**23**	1.432	1.393	**59**	1.437	1.393	**95**	1.441	1.390
**24**	1.372	1.450	**60**	1.367	1.450	**96**	1.439	1.409
**25**	1.422	1.396	**61**	1.427	1.396	**97**	1.408	1.386
**26**	1.392	1.402	**62**	1.387	1.402	**98**	1.395	1.438
**27**	1.422	1.392	**63**	1.426	1.392	**99**	1.393	1.386
**28**	1.371	1.432	**64**	1.369	1.432	**100**	1.417	1.419
**29**	1.433	1.390	**65**	1.436	1.390	**101**	1.375	1.374
**30**	1.407	1.406	**66**	1.404	1.406			
**31**	1.453	1.392	**67**	1.453	1.392			
**32**	1.436	1.438	**68**	1.436	1.448			
**33**	1.462	1.394	**69**	1.404	1.394			
**34**	1.406	1.407	**70**	1.453	1.407			
**35**	1.436	1.393	**71**	1.436	1.393			
**36**	1.368	1.450	**72**	1.461	1.450			

5.2 Transmission calculations.

Quantum transport calculations were conducted utilizing non-equilibrium Green's function (NEGF) by using the electronic structure of the junction obtained from Gaussian as the input for the Artaios package.[16] The junctions were formed by joining the previously relaxed geometries for the isolated molecule and the pyramidal gold contact with 20 Au atoms with the Sulfur-gold distance of 2.45 Å according to the literature.[17] We considered optimized geometry on both singlet and triplet configurations and performed single-point energy calculations using Gaussian at the B3LYP/Lanl2DZ level of theory as it has been suggested to minimize the ghost transmission and avoid extra through-space coupling between contacts.[16] Artaios software then extracted the Hamiltonian and the overlap matrices from the Gaussian output file, obtained from the previous step, and calculated the transmission in the wide-band limit. As a Local Density of States (LDOS) for the bulk gold electrodes, we used 0.036 eV^{-1} in agreement with the literature.[18]

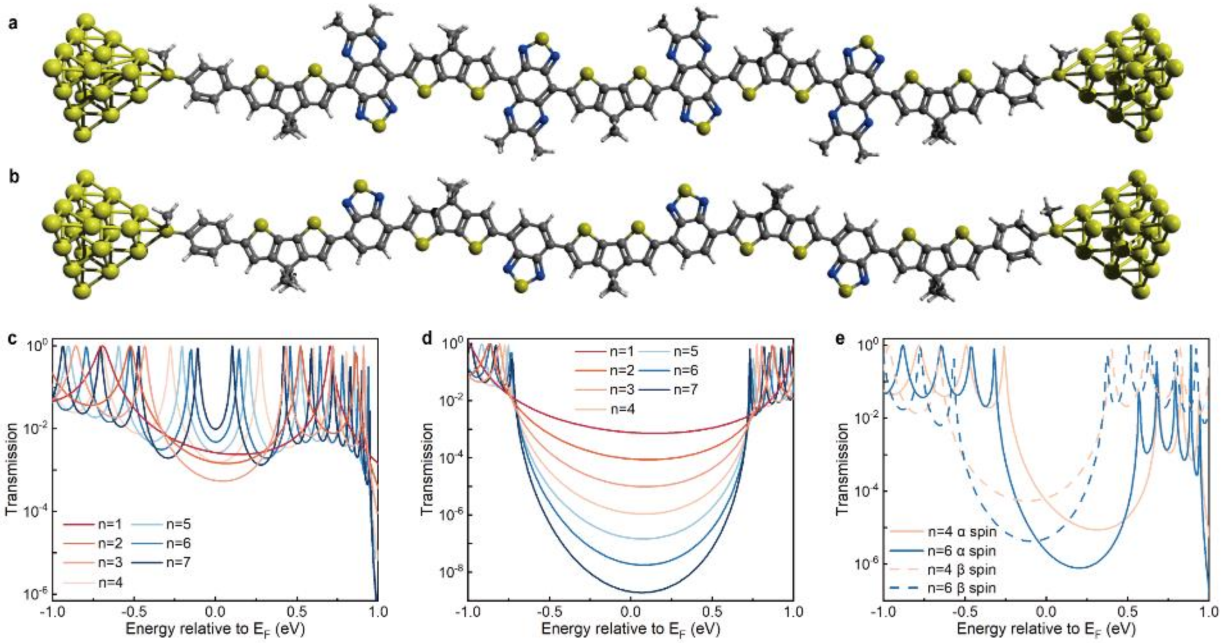

Figure S34. Structural geometry of a) open-shell and b) closed-shell control macromolecular junction with repeating unit of n = 4. Transmission functions calculated for c) open-shell macromolecules in singlet state and d) closed-shell control macromolecules with different numbers of repeating unit n. e) Transmission function for the open-shell macromolecules in the triplet state.

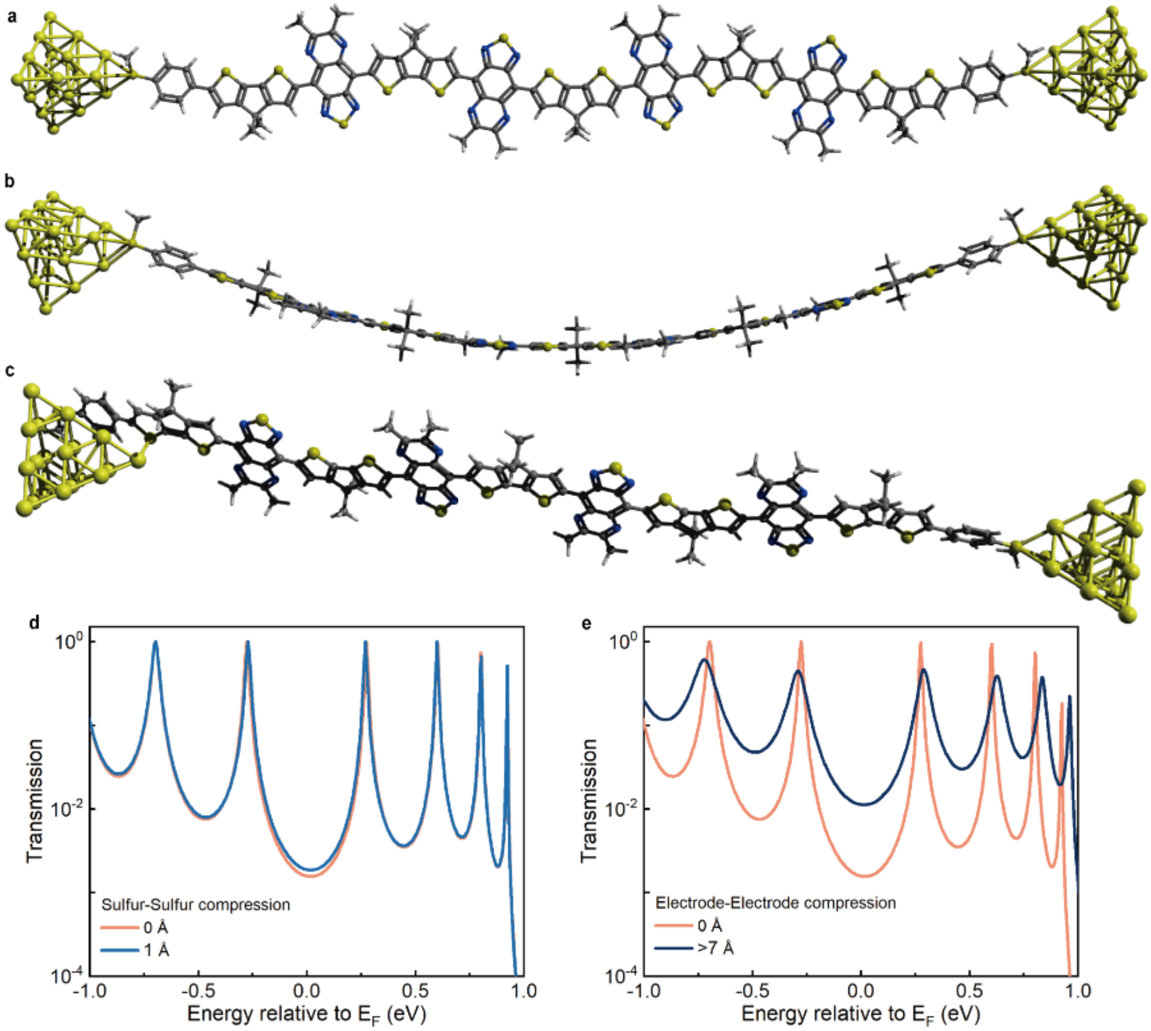

Figure S35. Transmission calculations for mechanically modulated macromolecule $n = 4$. a) side view and b) top view of the compressed molecule from sulfur atoms at both ends for 1 Å. At this step we first picked the relaxed geometry and moved the sulfurs at both ends towards each other by 1 Å and kept them fixed during the geometry optimization. c) schematic of the molecular junction compressed for more than 7 Å from the left electrode to bond with the next sulfur atom on the donor segment. At this step one of the electrodes was moved toward the other one until it bonded to the next sulfur atom. d) Transmission function calculated for both fully extended (red curve) and compressed (blue curve) macromolecule from sulfur atoms at both ends as shown in b. e) Transmission function calculated for both fully extended (red curve) and compressed (blue curve) macromolecule from the left electrode for more than 7 Å to bond to the next sulfur atom on the donor segment as shown in c.

6. References

[1] Madathil, P. K.; Cho, S.; Choi, S.; Kim, T.-D.; Lee, K.-S. Synthesis and Characterization of Cyclopentadithiophene and Thienothiophene-Based Polymers for Organic Thin-Film Transistors and Solar Cells. *Macromol. Res.* **2018**, *26*, 934-941.

[2] Ying, L.; Hsu, B. B. Y.; Zhan, H.; Welch, G. C.; Zalar, P.; Perez, L. A.; Kramer, E. J.; Nguyen, T.-Q.; Heeger, A. J.; Wong, W.-Y., et al. Regioregular Pyridal[2,1,3]thiadiazole π-Conjugated Copolymers *J. Am. Chem. Soc.* **2011**, *133*, 18538-18541.

[3] Susumu, K.; Duncan, T. V.; Therien, M. J. Potentiometric, Electronic Structural, and Ground- and Excited-State Optical Properties of Conjugated Bis[(Porphinato)zinc(II)] Compounds Featuring Proquinoidal Spacer Units. *J. Am. Chem. Soc.* **2005**, *127*, 5186-5195.

[4] Joo, Y.; Huang, L.; Eedugurala, N.; London, A. E.; Kumar, A.; Wong, B. M.; Boudouris, B. W.; Azoulay, J. D. Thermoelectric Performance of an Open-Shell Donor-Acceptor Conjugated Polymer Doped with a Radical-Containing Small Molecule. *Macromolecules.* **2018**, *51*, 3886-3894.

[5] Xu, B.; Tao, N. J. Measurement of single-molecule resistance by repeated formation of molecular junctions. *Science* **2003**, *301*, 1221-1223.

[6] Guo, C.; Wang, K.; Zerah-Harush, E.; Hamill, J.; Wang, B.; Dubi, Y.; Xu, B. Molecular rectifier composed of DNA with high rectification ratio enabled by intercalation. *Nat. Chem.* **2016**, *8*, 484-490.

[7] Quek, S. Y.; Kamenetska, M.; Steigerwald, M. L.; Choi, H. J.; Louie, S. G.; Hybertsen, M. S.; Neaton, J. B.; Venkataraman, L. Mechanically controlled binary conductance switching of a single-molecule junction. *Nat. Nanotechnol.* **2009**, *4*, 230-234.

[8] Li, L.; Low, J. Z.; Wilhelm, J.; Liao, G.; Gunasekaran, S.; Prindle, C. R.; Starr, R. L.; Golze, D.; Nuckolls, C.; Steigerwald, M. L.; Evers, F.; Campos, L. M.; Yin, X.; Venkataraman, L. Highly Conducting Single-Molecule Topological Insulators Based on Mono- and Di-Radical Cations. *Nat. Chem.* **2022**, *14*, 1061-1067.

[9] Li, L.; Louie, S.; Evans, A. M.; Meirzadeh, E.; Nuckolls, C.; Venkataraman, L. Topological Radical Pairs Produce Ultrahigh Conductance in Long Molecular Wires. *J. Am. Chem. Soc.* **2023**, *145*, 2492-2498.

[10] Zang, Y.; Fu, T.; Zou, Q.; Ng, F.; Li, H.; Steigerwald, M. L.; Nuckolls, C.; Venkataraman, L. Cumulene Wires Display Increasing Conductance with Increasing Length. *Nano Lett.* **2020**, *20*, 8415-8419.

[11] Leary, E.; Limburg, B.; Alanazy, A.; Sangtarash, S.; Grace, I.; Swada, K.; Esdaile, L. J.; Noori, M.; Gonzalez, M. T.; Rubio-Bollinger, G.; Sadeghi, H.; Hodgson, A.; Agraıt, N.; Higgins, S. J.; Lambert,

C. J.; Anderson, H. L.; Nichols, R. J. Bias-Driven Conductance Increase with Length in Porphyrin Tapes. *J. Am. Chem. Soc.* **2018**, *140*, 12877-12883.

[12] Capozzi, B.; Dell, E. J.; Berkelbach, T. C.; Reichman, D. R.; Venkataraman, L.; Campos, L. M. Length-Dependent Conductance of Oligothiophenes. *J. Am. Chem. Soc.* **2014**, *136*, 10486-10492.

[13] Zang, Y.; Ray, S.; Fung, E. D.; Borges, A.; Garner, M. H.; Steigerwald, M. L.; Solomon, G. C.; Patil, S.; Venkataraman, L. Resonant Transport in Single Diketopyrrolopyrrole Junctions. *J. Am. Chem. Soc.* **2018**, *140*, 13167-13170.

[14] Marongiu, M.; Ha, T.; Gil-Guerrero, S.; Garg, K.; Mandado, M.; Melle-Franco, M.; Diez-Perez, I.; Mateo-Alonso, A. Molecular graphene nanoribbon junctions. *J. Am. Chem. Soc.* **2024**, *146*, 3963-3973.

[15] Becke, A. D. A New Mixing of Hartree--Fock and Local Density-Functional Theories. *J. Chem. Phys.* **1993**, *98*, 1372-1377.

[16] Herrmann, C.; Solomon, G. C.; Subotnik, J. E.; Mujica, V.; Ratner, M. A. Ghost transmission: How large basis sets can make electron transport calculations worse. *J. Chem. Phys.* **2010**, *132*, 024103.

[17] Bilić, A.; Reimers, J. R.; Hush, N. S. The structure, energetics, and nature of the chemical bonding of phenylthiol adsorbed on the Au (111) surface: Implications for density-functional calculations of molecular-electronic conduction. *J. Chem. Phys.* **2005**, *122*, 094708-094723.

[18] Herrmann, C.; Solomon, G. C.; Ratner, M. A. Designing optimal organic spin filters in the coherent tunneling regime,. *J. Chem. Phys.* **2011**, *134*, 224306.
